# Five-year analysis of efficacy and safety of a bidirectional AAV gene therapy in Tay-Sachs sheep

**DOI:** 10.1172/JCI182942

**Published:** 2025-09-30

**Authors:** Toloo Taghian, Jillian Gallagher, Stephanie Bertrand, William C. Baker, Kalajan Lopez Mercado, Hector R. Benatti, Erin Hall, Yvette Lopez, Abigail McElroy, John T. McCarthy, Sanjana Pulaparthi, Deborah Fernau, Samuel Mather, Sophia Esteves, Elise Diffie, Amanda Gross, Hannah G. Lahey, Xuntian Jiang, Elizabeth Parsley, Rachael Gately, Rachel Prestigiacomo, Siauna Johnson, Amanda Taylor, Lindsey Bierfeldt, Susan Tuominen, Jennifer Koehler, Guangping Gao, Jun Xie, Qin Su, Robert King, Matthew J. Gounis, Vania Anagnostakou, Ajit Puri, Ana Rita Batista, Miguel Sena-Esteves, Douglas R. Martin, Heather Gray-Edwards

**Affiliations:** 1 Department of Radiology and; 2 Horae Gene Therapy Center, University of Massachusetts Chan Medical School, Worcester, Massachusetts, USA.; 3 Tufts Farm, Cummings College of Veterinary Medicine, Tufts University, North Grafton, Massachusetts, USA.; 4 Scott-Ritchey Research Center, Auburn University College of Veterinary Medicine, Auburn, Alabama, USA.; 5 Department of Medicine, Washington University, St. Louis, Missouri, USA.; 6 Department of Clinical Sciences,; 7 Department of Ambulatory and Theriogenology, and; 8 Tufts Field Service, Cummings School of Veterinary Medicine, Tufts University, North Grafton, Massachusetts, USA.; 9 Department of Clinical Sciences, Auburn University College of Veterinary Medicine, Auburn, Alabama, USA.; 10 Department of Animal Medicine, University of Massachusetts Chan Medical School, Worcester, Massachusetts, USA.; 11 Department of Pathobiology, Auburn University College of Veterinary Medicine, Auburn, Alabama, USA.

**Keywords:** Development, Therapeutics, Gene therapy, Lysosomes, Neurological disorders

## Abstract

Tay-Sachs disease (TSD) and Sandhoff disease are fatal neurodegenerative diseases without an effective therapy that are caused by mutations in the *HEXA* and *HEXB* genes, respectively. Together they encode the heterodimeric isozyme of hexosaminidase, hexosaminidase A (HexA), that degrades GM2 ganglioside. This report describes a 5-year-long study using a bidirectional adeno-associated virus 9 (AAV9) vector (AAV9-Bic_HexA/HexB) encoding both *HEXA* and *HEXB* in the TSD sheep model. Bidirectional AAV9 was delivered i.v. or through various cerebrospinal fluid (CSF) delivery routes: intracerebroventricular (ICV), cisterna magna (CM), and lumbar intrathecal space (LIT). The longest survival and best distribution were achieved by multipoint CSF delivery (combined CM, ICV, and LIT) with treated animals that survived up to 5 years of age (untreated animals with TSD die after ~9 months). Extension in survival was accompanied by lasting improvement in neurological examination and maze testing. Improvement in biomarkers of efficacy, including MRI, magnetic resonance spectroscopy, diffusion tensor imaging, and CSF levels of GM2 ganglioside and HexA activity, was evident. Postmortem assessments showed broad HexA distribution, GM2 ganglioside clearance, and vector genome distribution, especially in deep brain structures. Therapeutic efficacy documented in this study supports translation of bidirectional vector and multipoint CSF delivery to a clinical trial in patients with TSD and Sandhoff disease.

## Abstract 2

Multisite CSF delivery of bidirectional AAV gene therapy to treat Tay-Sachs sheep model results in dramatic CNS disease correction and survival up to 5 years

## Graphical abstract

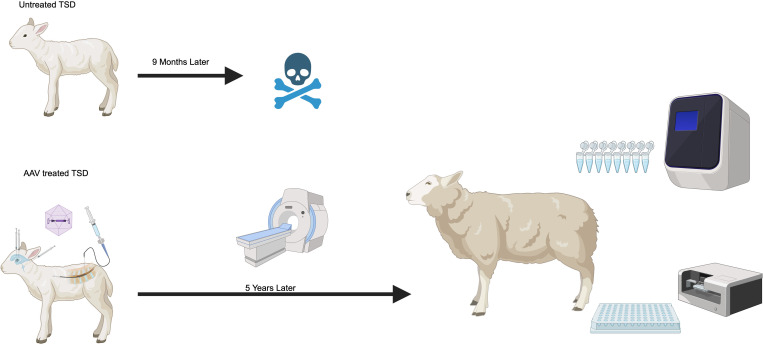

## Introduction

Tay-Sachs disease (TSD) and Sandhoff disease are characterized by progressive nervous system dysfunction and are classified according to disease severity as infantile, juvenile, or late onset. Children with the infantile form (the most severe) develop normally for the first 3–6 months of life, after which developmental regression occurs. By the age of 2 years, affected children suffer from frequent seizures, swallowing difficulties, respiratory infections, and loss of motor control. Death typically occurs before the fifth birthday (1). Juvenile-onset TSD is typically diagnosed around 4–6 years of age. Children develop learning, speech, and walking difficulties, which are followed by loss of learned skills, hypertonia, spasticity, and seizures. Patients eventually progress to a semi-vegetative state, and death frequently occurs in the second decade of life (2). Patients with late-onset TSD and late-onset Sandhoff disease have an onset of clinical signs that occurs in their early 20s, and most become wheelchair bound by 35–40 years of age. Clinical signs include ataxia, muscle weakness, abnormal saccades, swallowing and speech difficulties, peripheral neuropathy, and psychiatric disease (3). Currently there are no FDA-approved disease-modifying treatment options for TSD or Sandhoff disease, and management is limited to symptomatic treatment including anticonvulsants and psychiatric medications during a relentless progressive decline.

**Figure 1:**
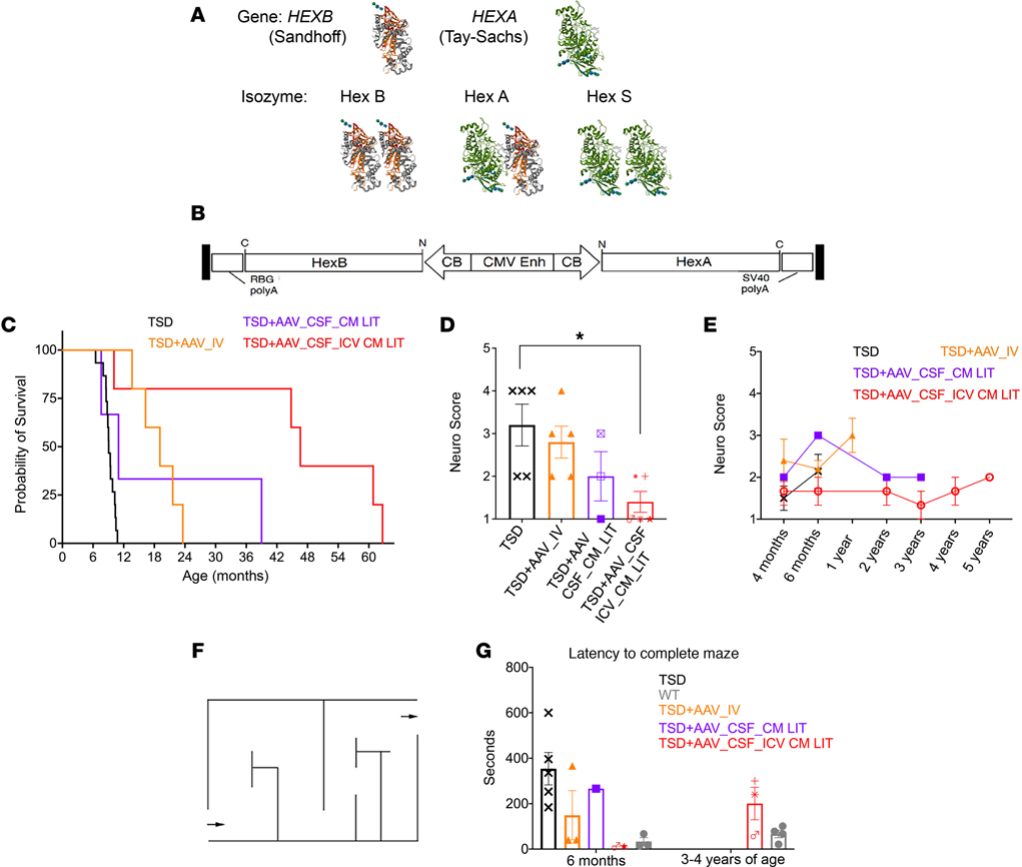
Hexosaminidase structure, AAV design, and improved survival and neurological scores by AAV treatment. (**A**) Hexosaminidase enzyme has 3 isozymes: HexA, HexB, and HexS. The HexA enzyme hydrolyzes GM2 ganglioside in humans and consists of α and β subunits, encoded by *HEXA* and *HEXB* genes, respectively. Deficiency in *HEXA* and *HEXB* genes results in TSD and Sandhoff disease, respectively. Hex α- and β-subunit protein x-ray diffraction structure was from the RCSB protein data bank (2GK1 PDB DOI: https//doi.org/10.22.10/pdb2GK1/pdb). (**B**) Schematics of bicistronic AAV9 vector expressing both *HEXA* and *HEXB* in a single AAV. (**C**) Kaplan-Meier plot of the survival rate of untreated sheep (*n* = 15) and bicistronic AAV9-treated TSD sheep (TSD+AAV_IV, *n* = 5; TSD+AAV_CSF_ICV-CM-LIT, *n* = 5; TSD+AAV_CSF_CM-LIT, *n* = 3). Survival of the TSD+AAV_IV and TSD+AAV_CSF_ICV-CM-LIT cohorts was significantly different than that of the TSD group (*P* < 0.001), while survival of TSD+AAV_CSF_CM-LIT cohort was not different than the TSD group (*P* = 0.059). The log-rank test was used for statistical analysis between cohorts. (**D**) The neurological score of sheep was described by scoring from 1 (normal) to 5 (worse). The TSD+AAV_CSF_ICV-CM-LIT cohort had significantly decreased scores compared with untreated TSD sheep (*P < 0.05). Scores were analyzed by Mann-Whitney tests, and effect was determined by a Hodges-Lehmann test. The neurological scores were collected at the time of euthanasia, corresponding to the survival curve in **C**. (**E**) Total neurological score of sheep over time. Statistical analyses were not completed for these data because some time points only had 1 recording per group (TSD, *n* = 3; TSD+AAV_IV, *n* = 5; TSD+AAV_CSF_ICV-CM-LIT, *n* = 3 up to year 4, and *n* = 1 at 5 years; TSD+AAV_CSF_CM-LIT, *n* = 1). (**F**) Schematic of the maze used to test sheep cognition. (**G**) Bar chart showing latency to complete the maze for all sheep at 6 months or at 3–4 years of age (6 months: TSD *n* = 5, WT *n* = 3, TSD+AAV_IV *n* = 3, TSD+AAV_CSF-CM-LIT *n* = 2, TSD+AAV_CSF_ICV-CM-LIT *n* = 2; 3–4 years: TSD+AAV_ CSF_ICV-CM-LIT *n* = 3, WT *n* = 4).

TSD and Sandhoff disease are lysosomal storage disorders caused by mutations in the *HEXA* and *HEXB* genes, encoding the α and β subunit, respectively. β-N-acetyl-d-hexosaminidase A (HexA) (enzyme class 3.2.1.52) consists of a heterodimeric complex of α and β subunits; therefore, mutations in either gene result in reduced enzymatic activity (4). HexA deficiency leads to progressive accumulation of GM2 ganglioside with ensuing neurodegeneration in the central and peripheral nervous systems; severity inversely correlates with residual enzymatic activity. GM2 ganglioside is degraded by the coordinated action of HexA and the GM2 activator protein, a nondegradative accessory protein necessary for GM2 ganglioside presentation to HexA. Hex α and β subunits can also self-dimerize to form separate isozymes with different substrate specificities: HexB (ββ) and HexS (αα) (an unstable isoform present at very low levels) (Figure 1A), but only HexA is capable of GM2-ganglioside degradation in humans (5).

Adeno-associated virus (AAV) vectors have emerged as the most potent platform for efficient and stable in vivo gene transfer to the CNS with minimal or absent intrinsic toxicity. AAV vectors have become the platform of choice for development of effective therapies for neurological diseases, and some clinical trials have reported encouraging (6) or even transformative results (7). Intracranial co-administration of 2 monocistronic vectors encoding the human transgene in Sandhoff disease mice restored the enzyme activity, corrected neuropathology, and extended lifespan (8–10). Similar results were observed in feline models of Sandhoff disease (11–14). Similarly, TSD sheep exhibited normalization of storage and neuroinflammation in the brain after intracranial injection of monocistronic vectors (15). This dual-vector AAV gene therapy approach has been tested in 2 patients with infantile TSD under an expanded access investigational new drug (application 18225) and a recently concluded phase I/II study (ClinicalTrials.gov NCT04669535) (16, 17).

While thalamic injections have been shown to be highly effective for widespread enzyme distribution in the cerebrum with encouraging preliminary data in patients, the thalamus is a high-risk target because of its critical functions as an integration and relay center. Therefore, we developed a CSF-based approach using a bicistronic AAV9 vector (AAV9-Bic_HexA/HexB) expressing both *HEXA* and *HEXB* in a single AAV to enable coexpression of both subunits in each transduced cell. The therapeutic efficacy of this AAV9 vector was tested in Sandhoff disease mice by i.v. delivery showing significant extension of lifespan and normalization of neurochemistry (18). Here we report the therapeutic efficacy results in 5-year-old TSD sheep with a similar bicistronic AAV9 vector expressing ovine *HEXA/HEXB* cDNAs delivered by i.v. delivery or CSF infusion. These results support the translation of this AAV9-based CSF gene therapy for patients with juvenile and late-onset TSD and Sandhoff disease.

## Results

### Summary of cohorts.

This study had 4 treatment groups (Table 1) and 2 control groups: (a) TSD sheep treated by i.v. injection with 5 × 10^13^ viral genome (vg)/kg at 9–13 days of age (*n* = 5). (b) TSD sheep treated by CSF delivery via 4 sites: bilaterally in the lateral ventricles (ICVs), cisterna magna (CM), and the lumbar intrathecal (LIT) space, 1 × 10^14^ vg total dose at 3 weeks of age, 75% divided between ventricles, 25% divided between the LIT and CM (TSD+AAV_CSF_ICV-CM-LIT; *n* = 4 animals harvested at 4 months after injection for biochemical assessment; *n* = 5 for long-term survival and clinical benefit and durability). The total AAV9 dose was approximately equivalent across treated cohorts when comparing doses by body weight. (c) TSD sheep treated by CSF delivery at 3 weeks of age with 1 × 10^14^ vg administered at the CM (75%) and LIT (25%) (TSD+AAV_CSF_CM-LIT; *n* = 3. (d) Untreated TSD controls (*n* = 15). Also included were (e) WT sheep controls (*n* = 10). Intravenous delivery was performed at an earlier age to minimize the total AAV required while maintaining an equivalent high dose.

### Survival.

Long-term survival and therapeutic benefit as measured by neurological evaluation were as follows (Figure 1, C–E). The 4-site, CSF-treated cohort (TSD+AAV_CSF_ICV-CM-LIT) (represented by the red line in Figure 1, C and E) had an average survival of 45 (± 21.1 SD) months, with the oldest animal at 62.7 months (>5 years of age). Supplemental Videos 1, A and B show WT and untreated TSD as compared with Supplemental Videos 1, C and D for 2 oldest treated sheep, 212 and 271. TSD+AAV_CSF_ICV-CM-LIT animals had an average proprioceptive neuro score at endpoint of 1.4 out of 5 (5 being worst) throughout the study and were euthanized due to cervical trauma (from ramming by a herd mate), heart failure, or ruptured metacarpal collateral ligament. Sheep treated by CM and LIT delivery only (TSD+AAV_CSF_CM-LIT; purple line) survived an average of 19.2 (± 17.2 SD) months. Compared with animals treated in all CSF sites, TSD+AAV_CSF_CM-LIT sheep had lower mean scores on neurological exams over time, although differences were not statistically significant, with euthanasia due to aspiration pneumonia, ataxia, weight loss, or blindness. The intravenous AAV-treated cohort (TSD+AAV_IV) (represented by the orange line in Figure 1C) had an average survival of 18.9 (± 3.9 SD) months. TSD+AAV_IV animals had a neurological exam score of 3 out of 5 at endpoint, and animals were all euthanized due to distal limb tendon or ligament rupture, which is not consistent with the natural history of TSD in sheep. All treated sheep were evaluated at necropsy for evidence of hepatocellular carcinoma or any other abnormalities consistent with neoplasia or immune response, for which no evidence was found. Untreated TSD sheep (indicated by the black line in Figure 1, C and E, and the black box in Figure 1B) had an average neuro score of 3 out of 5 and a survival of 9.4 (± 0.8 SD) months, with euthanasia due to ataxia and falling.

### Behavioral testing.

To determine level of functional rescue, TSD sheep were put through a maze with completion time recorded (Figure 1, F and G, and Supplemental Videos 2, A–D; supplemental material available online with this article; https://doi.org/10.1172/JCI182942DS1). The flock was located out of sight in a holding pen at the end of the maze but were clearly audible to the test subject; thus, the strong flocking instinct of sheep was used as motivation to complete the maze. Audition was retained in the TSD sheep, and brain-stem auditory evoked responses were within normal limits at the time of humane endpoint (19). Untreated TSD sheep at 6 months of age (6–7 months of age; *n* = 4) (indicated by black Xs in Figure 1G) took 353 (± 159 SD) seconds longer to complete the maze compared with WT controls (*n* = 5) (gray circles in Figure 1G). Decreased time to navigate the maze was noted in the TSD+AAV_CSF_ICV-CM-LIT group at 6 months, with increased time to completion at 3–4 years of age. Intermediate improvement was noted in the TSD+AAV_IV cohort at 6 months of age; we note that the slowest animal was nearing humane endpoint at the time of the exam. The TSD+AAV_CSF_CM-LIT sheep completed the maze at a time equivalent to that of TSD control sheep.

### CSF HexA and GM2 levels.

**Figure 2:**
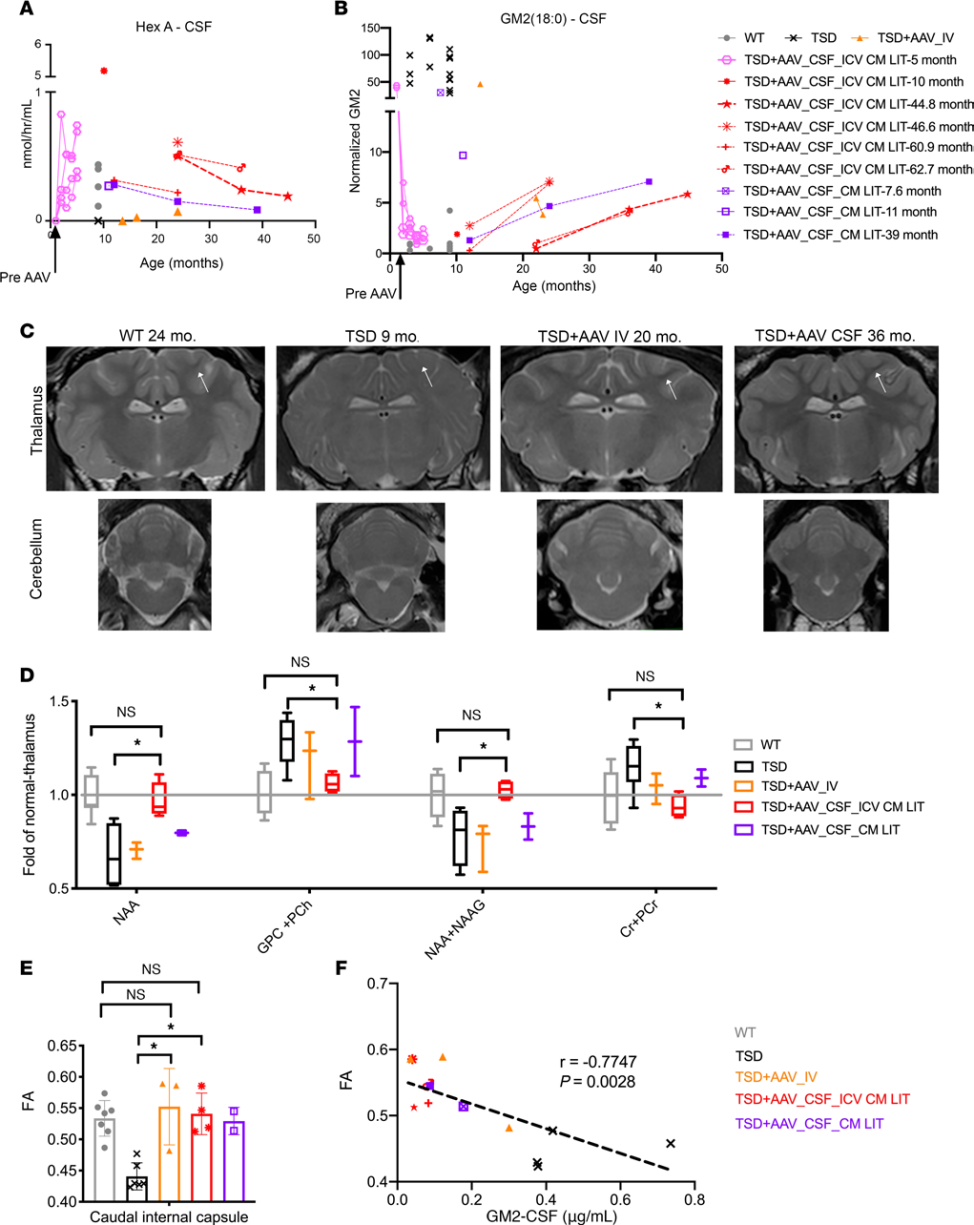
Noninvasive monitoring of bicistronic AAV9-treated TSD sheep. (**A** and **B**) HexA in CSF of short-term-treated sheep (*n* = 4 TSD+AAV_CSF-ICV-CM-LIT; pink hexagon-solid lines) shows a sharp increase to at least WT levels by 1 month after AAV administration (*n* = 10; gray circles). Similarly, there is a sharp decrease in GM2 levels into the normal range by 1 month after treatment. In the long-term-treated cohort (TSD+AAV_CSF-ICV-CM-LIT; red-dotted lines), GM2 shows a steady increase accompanied by an inversely proportional decrease in HexA levels approximately 1 year after AAV administration (each red symbol represents 1 sheep in the long-term cohort). Analyzed TSD (*n* = 14; black crosses). (**C**) T2-weighted MRI of sheep thalamus and cerebellum demonstrate normalization of white matter intensity (white arrow) in both TSD+AAV_IV and TSD+AAV_CSF_ICV-CM-LIT treated cohorts. (**D**) MRS in sheep thalamus indicates the normalization (no statistical difference from WT; *n* = 7) of markers of neuronal health, demyelination, and energy metabolism for the TSD+AAV_CSF_ICV-CM-LIT group (*n* = 4) and significantly different levels as compared with TSD sheep (*P < 0.05; *n* = 8 TSD sheep). TSD+AAV_IV (*n* = 3) and TSD+AAV_CSF_CM-LIT (*n* = 2) groups show intermediate levels. Brown-Forsythe and Welch’s ANOVA tests followed by Dunnett’s T3 test were performed. The data shown for MRS and DTI analysis in this figure represent 1-time imaging of each sheep at the endpoint. NAA, GPC + PCh, NAA+NAAG, Cr+PCr. (**E**) DTI scalar FA, which informs about the degree of anisotropy in diffusion of water in white matter, decreases significantly in TSD sheep as compared with WT sheep in the caudal internal capsule. AAV_IV and CSF cohorts show normalization on FA about 2–3 years after AAV administration (*P < 0.05). The Kruskal-Wallis test followed by Dunn’s test was performed. (**F**) FA scalar shows a negative and significant association with GM2 levels in sheep CSF (*P* = 0.0028). Spearman’s test was used for correlation analysis.

HexA activity and GM2 ganglioside levels were quantified in the CSF and serum (HexA only) of untreated TSD sheep, WT controls, and all AAV-treated cohorts (Figure 2, A and B, and Supplemental Figure 1A). As expected, untreated TSD sheep had very low HexA and elevated GM2 levels in CSF compared with WT controls. In the TSD+AAV_CSF_ICV-CM-LIT cohort, HexA activity was at or above normal levels (range, 0.5- to 2.7-fold of normal) and GM2 level was normalized by 1 month after treatment (similar levels as WT). HexA activity remained in the normal range in all samples. Later time points in the TSD+AAV_CSF_ICV-CM-LIT and TSD+AAV_CSF_CM-LIT animals trended toward decreased HexA and increased GM2 concentrations at 2–4 years after gene therapy. The TSD+AAV_IV cohort had lowest HexA levels (8%–20% of normal) and elevated GM2 levels in CSF. HexA in serum remained at the levels of untreated TSD sheep in all AAV-treated cohorts after gene therapy (Supplemental Figure 1A).

### MRI-based metrics.

To determine the degree of normalization in the brain after gene therapy, MRI was performed. T2-weighted MRI revealed normalization of gray and white matter intensities in i.v.- or CSF-treated sheep at 20 or 36 months of age, respectively, compared with untreated TSD sheep at 9 months (humane endpoint) (Figure 2C). To monitor brain biochemistry, MR spectroscopy (MRS) was performed (Figure 2D). In TSD+AAV_CSF-ICV-CM-LIT animals, there was normalization in the thalamus for markers of neuron and axon health (namely, *N*-acetylaspartate [NAA] and *N*-acetylaspartylglutamate [NAAG]), myelination (glycerophosphocholine [GPC] plus phosphocholine [PCh]) and metabolism (creatine [Cr] plus phosphocreatine [PCr]) compared with untreated controls. Intermediate improvement was noted in the TSD+AAV_CSF-ICV-CM-LIT and TSD+AAV_IV groups. Diffusion tensor imaging (DTI) was performed to further evaluate integrity of myelination (Figure 2, E and F, and Supplemental Figure 1, B and C), with normalization of fractional anisotropy (FA) (Figure 2E) and radial diffusivity (RD) (Supplemental Figure 1, B and C) in all AAV-treated cohorts. Interestingly, both FA and RD strongly correlated with GM2 levels in the CSF.

### HexA enzymatic assays, GM2 storage clearance, and biodistribution.

**Figure 3:**
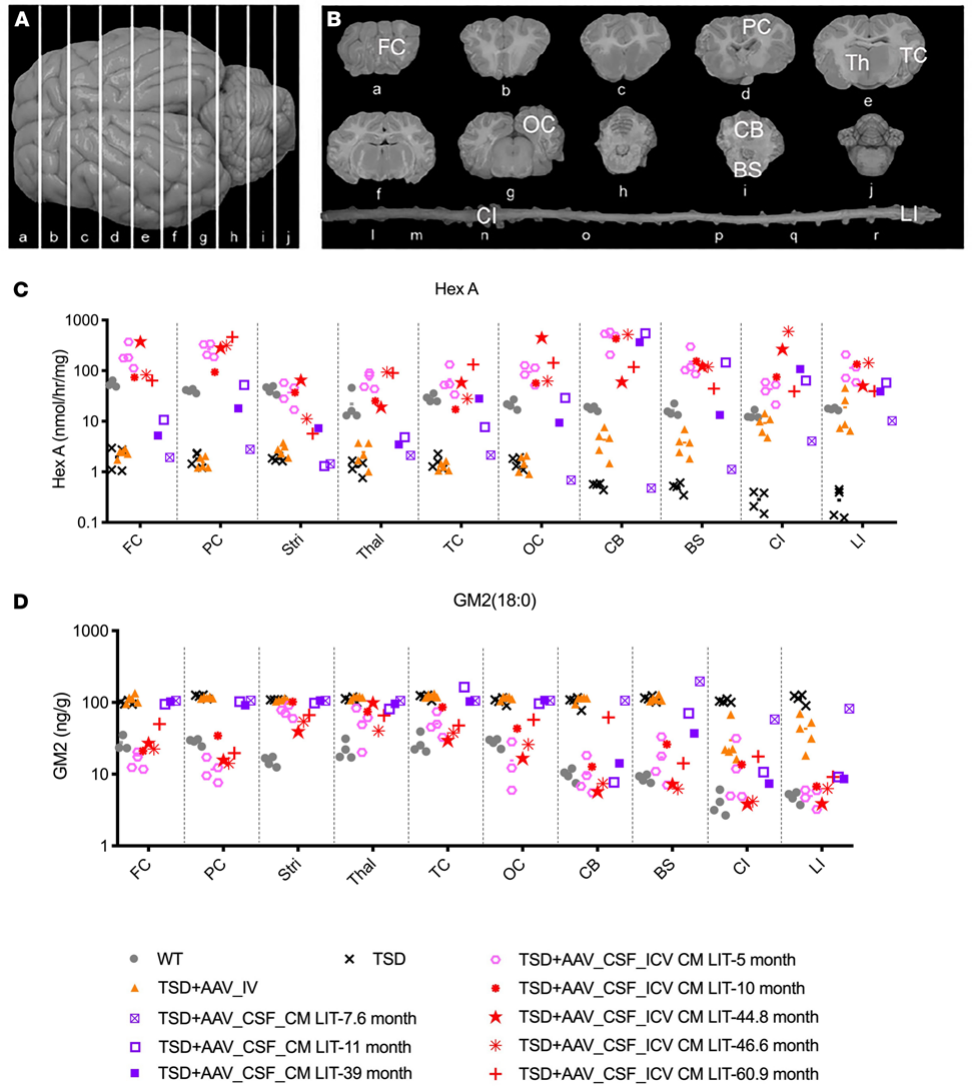
HexA activity and GM2 ganglioside levels in CNS after bicistronic AAV administration. (**A**) Dorsoventral view of the sheep brain, with white lines showing corresponding brain sections. (**B**) Analyzed regions include frontal cortex (FC), parietal cortex (PC), thalamus (Th), temporal cortex (TC), occipital cortex (OC), cerebellum (CB), brainstem (BS), cervical intumescence (CI), and lumbar intumescence (LI). (**C**) HexA activity. Short-term TSD+AAV_CSF_ICV-CM-LIT cohort (pink hexagon) had HexA levels similar to or higher than WT (gray circles) levels in all analyzed CNS regions and were not significantly different from WT at 5 months. The TSD+AAV_IV cohort had significantly less HexA levels in all analyzed region as compared with WT except for Thal, CI, and LI. (**D**) GM2 ganglioside. Short-term TSD+AAV_CSF_ICV-CM-LIT cohort (pink hexagon) had HexA levels similar in same range as WT (gray circles) levels in all analyzed CNS regions and were not significantly different from WT at 5 months except for striatum. TSD+AAV_IV cohort had significantly higher GM2 levels as compared with WT (not significantly different from TSD) in all analyzed regions except for CI and LI, where no statistical difference was obtained as compared with WT (note: this might be due to variability in data). Brown-Forsythe and Welch’s ANOVA tests followed by Dunnett’s T3 test were performed for statistical analysis in **C** and **D**. Assays were repeated at least 3 times.

Throughout the CNS, including deep brain structures, HexA activity was restored to normal or above normal levels in the TSD+AAV_CSF_ICV-CM-LIT cohort 4 months after treatment, and levels remained in this range after approximately 4 years (Figure 3, A–D). In the TSD+AAV_CSF_CM-LIT cohort at endpoint, HexA activity trended lower in the forebrain (di- and telencephalon) as compared with TSD+AAV_CSF-ICV-CM-LIT animals, especially in the thalamus and frontal cortex. In the TSD+AAV_IV cohort at endpoint, HexA activity was low (near untreated TSD sheep levels) in the forebrain, but HexA activity increased in the hindbrain (cerebellum and medulla) and spinal cord (Figure 3C). As expected, areas with higher-than-normal HexA activity showed the best GM2 storage clearance. In the TSD+AAV_CSF-ICV-CM-LIT group, GM2 was approximately in the normal range, with little to no GM2 clearance in the IV cohort (Figure 3D).

Biodistribution of vector genomes was in line with HexA activity and GM2 reduction in all cohorts (Supplemental Figure 2A). One treated sheep in the TSD+AAV_CSF_CM-LIT cohort did not benefit from the treatment and was euthanized at 7.6 months. We acknowledge the possibility that the catheter and guide wire created a hole in the dura during AAV delivery, which could have allowed for vector leakage into the epidural space; however, no evidence of this was noted intraoperatively.

**Figure 4:**
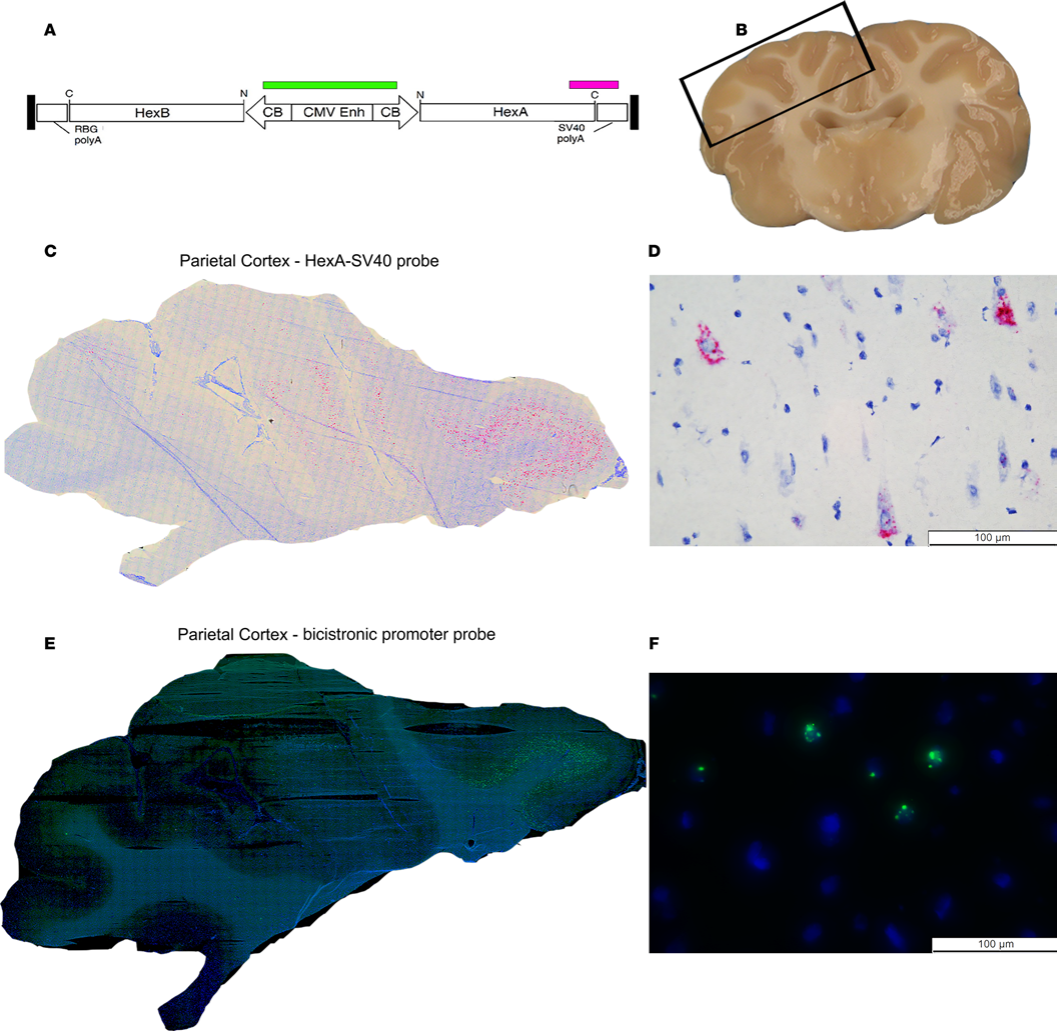
Vector biodistribution assessment by RNAscope and BaseScope. (**A**) HexA-SV40 probe (BaseScope, pink) and bicistronic promoter probe (RNAscope, green). (**B**) Gross location of the parietal cortex of the TSD+AAV_CSF-ICV-CM-LIT at 5 months of age. Representative photomicrographs of HexA-SV40 probe (**C** and **D**). and bicistronic promoter probe (**E** and **F**) in the parietal cortex.

To further evaluate biodistribution, RNAscope targeting the promoter in the vector genome and Basescope targeting the 3′ end of HexA and 5′ end of the polyA were used. As expected, the 2 in situ probes highly correlated across in the parietal cortex of the short-term (5 months of age) TSD+AAV_CSF_ICV-CM-LIT cohort and appeared primarily neuronal based on morphological examination (Figure 4). Expression in glial cells appeared to be below our level of detection. Similar analyses were performed using RNAscope in the frontal cortex of a long-term treated TSD+AAV_CSF_ICV-CM-LIT cohort at 60.9 months of age. In line with HexA and qPCR analyses, biodistribution of the AAV vector in the frontal cortex of the long-term-treated sheep was in line with RNAscope results (Supplemental Figure 3).

**Figure 5:**
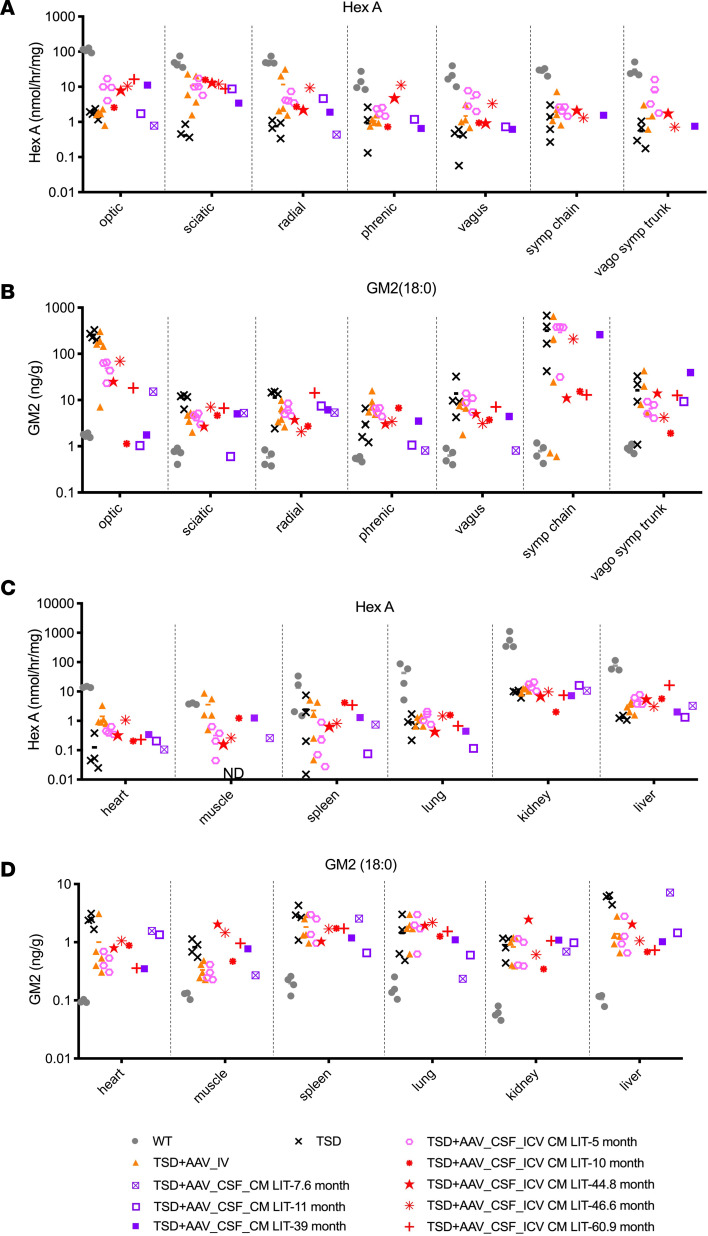
HexA activity and GM2 ganglioside levels in the nerves and peripheral organs after bicistronic AAV administration. (**A**) HexA activity for nerves in both TSD+AAV_IV and short-term TSD+AAV_CSF_ICV-CM-LIT (pink hexagons) cohorts showed an increase from that of the TSD controls. This was most notable for the optic, sciatic, and vagosympathetic trunk nerves; however, HexA expression in all analyzed nerves was not significantly different from the TSD controls. (**B**) GM2 ganglioside levels in nerves remained statistically the same as in the TSD group for both TSD+AAV_IV and short-term TSD+AAV_CSF_ICV-CM-LIT cohorts (pink hexagons) except for optic nerve in the TSD+AAV_CSF_ICV-CM-LIT cohort, which was different than in the TSD cohort (*P < 0.02). (**C**) HexA activity in peripheral organs remained similar to that of the TSD cohort for TSD+AAV_IV and short-term TSD+AAV_CSF_ICV-CM-LIT (pink hexagons) cohorts except for muscle. HexA was not detectable in TSD muscle. HexA in skeletal isolated from the TSD+AAV_IV group was not different from WT, while in the short-term TSD+AAV_CSF_ICV-CM-LIT (pink hexagons), HexA activity was significantly higher than in WT. (**D**) Levels of GM2 ganglioside in peripheral organs, except for heart and liver, remained the same as in the TSD group for both the TSD+AAV_IV and short-term TSD+AAV_CSF_ICV-CM-LIT (pink hexagons) cohorts. GM2 ganglioside levels in hearts of the short-term TSD+AAV_CSF_ICV-CM-LIT sheep were significantly lower than TSD levels (*P < 0.04). Similarly, for livers in the TSD+AAV_IV cohort, GM2 levels were significantly less than in the TSD group (*P < 0.05). Brown-Forsythe and Welch’s ANOVA tests followed by Dunnett’s T3 test were performed for statistical analysis in **A**–**D**. Assays were repeated at least 3 times. Symp chain, sympathetic chain; vago symp trunk, vagosympathetic trunk.

In nerves, HexA activity and GM2 clearance in all groups were roughly equivalent and intermediate of WT and TSD sheep, except for the optic nerve, where the TSD+AAV_CSF_ICV-CM-LIT group trended higher in HexA (Figure 5, A and B). Partial GM2 clearance was only appreciated in the optic nerve. Vector biodistribution mimicked HexA activity and GM2 clearance (Supplemental Figure 2B). In the peripheral organs, HexA expression was above untreated levels only in the heart and skeletal muscle, with the TSD+AAV_IV cohort trending higher than the TSD+AAV_CSF_ICV-CM-LIT or TSD+AAV_CSF_ CM-LIT groups (Figure 5C). Similarly, vector biodistribution also trended higher for the TSD+AAV_IV cohort as compared with the TSD+AAV_CSF_ICV-CM-LIT or TSD+AAV_CSF_ CM-LIT groups (Supplemental Figure 2C). Lastly, GM2 storage levels were in line with HexA expression in peripheral organs (Figure 5D).

Because most of the AAV-treated sheep in the study had to be euthanized due to tendon and/or ligament failure, we investigated if this could be due to an off-target effect of gene therapy. We evaluated HexA and total hexosaminidase activity of the AAV_IV animals because they had the greatest AAV dose directed to the periphery. Hexosaminidase activities in AAV-treated sheep were not significantly different from that in WT control sheep in tendons. Additionally, vector genomes were not detectable in tendons of the AAV_IV cohort (Supplemental Figure 4).

### Impact of preexisting immunity on HexA expression.

Due to cohousing of sheep after the TSD+AAV_IV cohort was treated, all the short-term animals in the TSD+AAV_CSF-ICV-CM-LIT cohort developed preexisting anti-AAV9 neutralizing antibodies (NAbs) in the blood (titer range, 1:10 to 1:20 (data not shown) and CSF (1:10) by the time of treatment. Interestingly, the animals with the fastest rise in anti-AAV9 titer in CSF had the lowest HexA increase in CSF over time (Supplemental Figure 5). All the long-term-treated CSF animals also had NAb titers ranging from 1:10 to 1:20 at the time of treatment.

### Qualitative histologic evaluation of the CNS.

**Figure 6:**
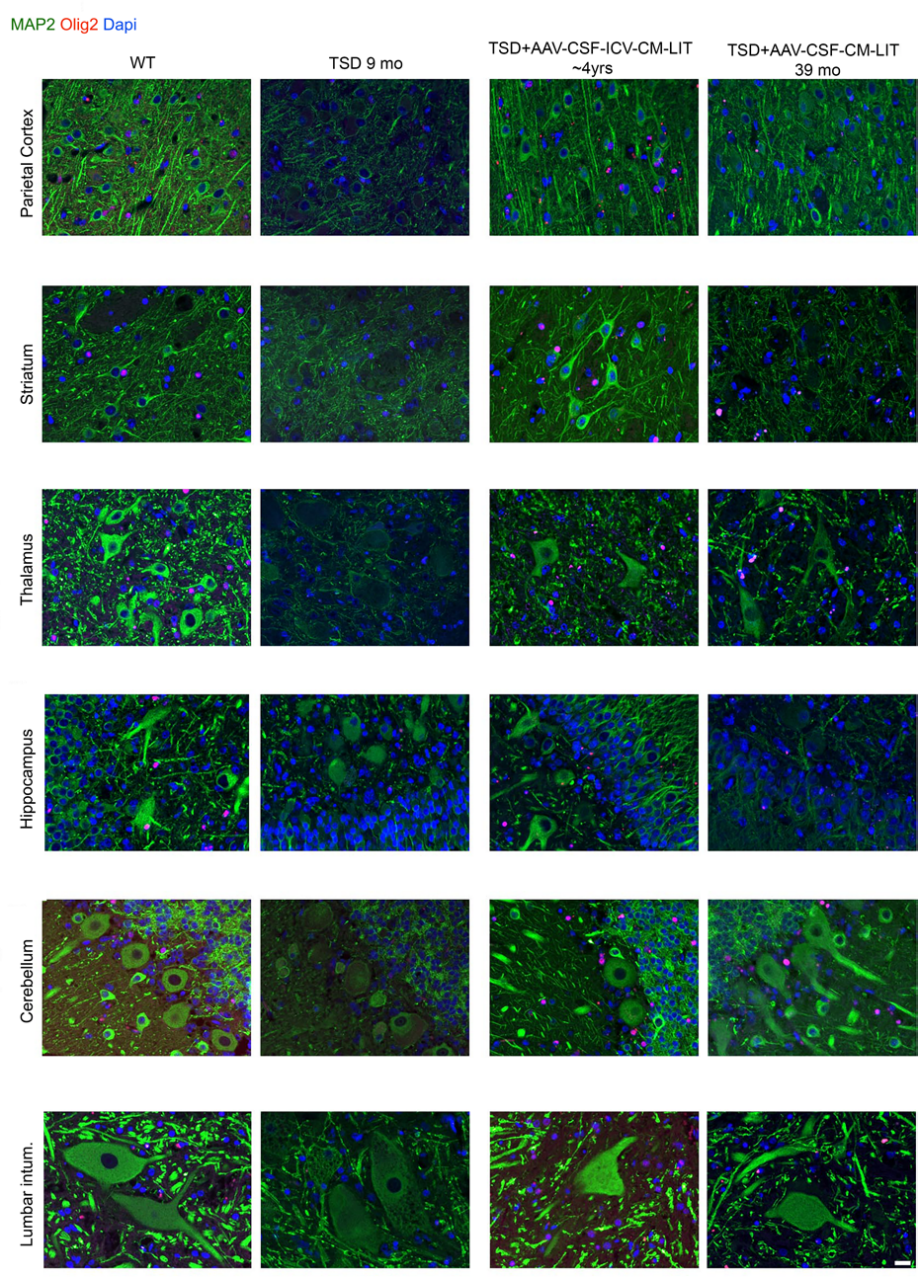
Immunofluorescence evaluation of neurons and oligodendrocytes in sheep CNS up to 4 years after AAV administration. MAP2, Olig2, and DAPI staining. Scale bar: 20 μm. Intum, intumescence.

**Figure 7:**
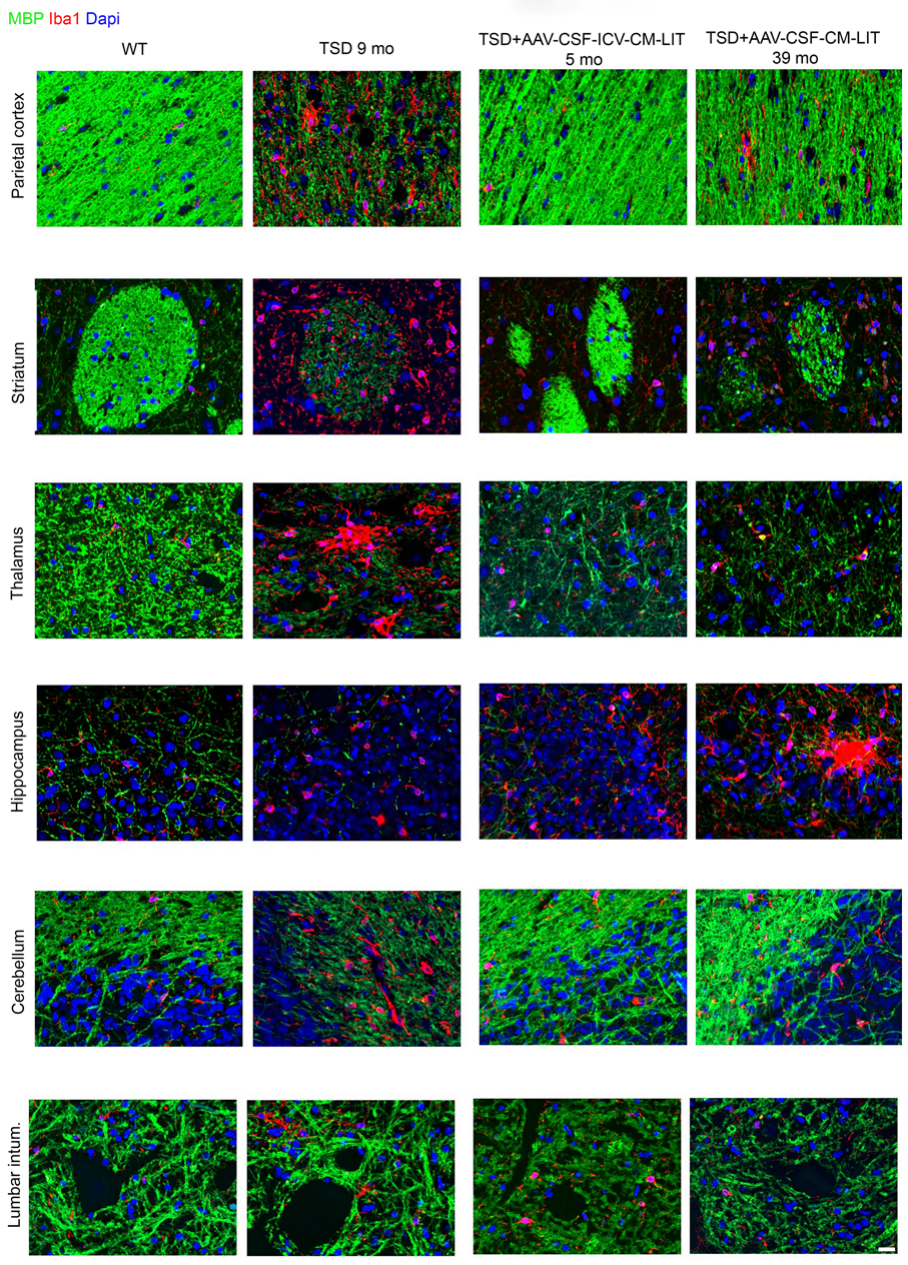
Immunofluorescence evaluation of white matter and glia in sheep CNS up to 4 years after AAV administration. MBP, calcium-binding adapter molecule 1 (Iba1), and DAPI staining. Scale bar: 20 μm. Intum, intumescence.

On H&E staining, neuronal morphology was normalized throughout the CNS in both the short-term and approximately 4-year-old TSD+AAV_CSF_ICV-CM-LIT sheep as compared with the widespread neuronal vacuolation of the untreated TSD sheep (Supplemental Figure 6). In the TSD+AAV_CSF_CM-LIT cohort, we observed correction of the spinal cord and cerebellum, but vacuolation persisted in the forebrain cortical and deep brain structures (Supplemental Figure 6). Immunofluorescent staining of neurons (microtubule-associated protein 2 [MAP2]) and oligodendrocytes (olig2) qualitatively showed normal oligodendrocyte and neuropil staining by 5 months after treatment that persisted until at least 4 years after AAV gene transfer in the TSD+AAV_CSF_ICV-CM-LIT group (Figure 6 and Supplemental Figure 7 for additional evaluated regions). In the TSD+AAV_CSF_CM-LIT group, appropriate oligodendrocyte and neuronal morphologies were observed in the spinal cord and cerebellum and to some extent in cortical structures, but not in the thalamus, striatum, or hippocampus (Figure 6 and Supplemental Figure 7 for additional evaluated regions). Myelination, as measured by myelin basic protein (MBP), was restored in all structures in the TSD+AAV_CSF-ICV-CM-LIT cohort at all ages, except with some loss of myelination after 4 years in the thalamus and hippocampus (Figure 7 and Supplemental Figure 8 for additional evaluated regions). The striatum, thalamus, and hippocampus of the TSD+AAV_CSF_CM-LIT cohort had qualitatively less myelin than the TSD+AAV_CSF_ICV_CM-LIT cohort. Microgliosis was attenuated in the TSD+AAV_CSF_ICV-CM-LIT cohort in all brain areas except the hippocampus (Figure 7 and Supplemental Figure 8 for additional evaluated regions). In the TSD+AAV_CSF_CM-LIT cohort, amoeboid activated microglia were evident in the cortex, thalamus, and hippocampus, but otherwise were ramified and in appropriate numbers elsewhere in the CNS. Astrocytosis as measured by glial fibrillary acidic protein immunostaining in the TSD+AAV_CSF_ICV_CM-LIT cohort was like that in the WT control at all ages but progressed like untreated TSD in the forebrain of the TSD+AAV_CSF_CM_LIT sheep 39 months after treatment (Supplemental Figure 9).

### Dorsal root ganglia.

One TSD+AAV_CSF_ICV-CM-LIT sheep died at 10 months of age from a fracture of the cervical vertebrae at C1–C2 due to ramming by a herd mate. This animal was found immediately, and a necropsy was performed. Dorsal root ganglia harvested at the level of cervical and lumbar intumescence showed no histopathological alterations (Supplemental Figure 10A). In fact, neuronal morphology was markedly improved from that of untreated TSD sheep that reached humane endpoint at a similar age (9 months). HexA activity in dorsal root ganglia from all groups was intermediate of WT control and untreated TSD sheep (Supplemental Figure 10, B and C).

## Discussion

Infantile TSD and Sandhoff disease are severe pediatric neurological diseases. The single most effective treatment to date for TSD and Sandhoff disease has been the placement of gastric feeding tubes, which extends the life of patients in a semi-vegetative state. Bone marrow transplant and enzyme replacement therapy have been tried, but both had minimal impact on the neurological component of the disease (20–22). Our team previously developed a dual monocistronic AAV gene therapy system to treat patients with TSD and Sandhoff disease, with an expanded access (*n* = 2) protocol followed by a recently completed phase I/II clinical trial (ClinicalTrials.gov NCT04669535) (16). This monocistronic AAV vector strategy showed minimal benefit in the TSD sheep model (survival to ~13 months after AAV) (15) but dramatic improvement in the cat and mouse Sandhoff disease models (8–12, 14). We hypothesize the difference in efficacy is due to size of the brain and CNS. The monocistronic vector strategy in patients with infantile TSD has reported early positive effects including disease stabilization, myelination on MRI, improved social interaction, and prolonged oral feeding (16). Here we describe superior efficacy in TSD sheep, using a single-vector design encoding both *HEXA* and *HEXB* genes. Because the response to gene therapy in TSD sheep was dramatically better with AAV9-Bic_HexA/HexB, we anticipate an improved response will translate to patients. Additionally, this new single AAV vector encodes the same *HEXA/HEXB* cDNA sequences; simplifies the chemistry, manufacturing, and controls required for drug development; and lowers the cost. The results reported here in TSD sheep, and previously in Sandhoff disease mice, support translation of this bicistronic AAV9 to the clinic, and it will be a second-generation AAV gene therapy for GM2 gangliosidosis.

Normal HexA heterodimer formation is driven by the endogenous levels of the α subunit relative to the β subunit (9, 23). The α and β subunit precursor proteins are translated and translocated into the lumen of the ER, where they dimerize to form immature HexA and HexB molecules (24, 25). In our first-generation TSD gene therapy, we used 2 monocistronic AAVrh8 vectors encoding HexA and HexB proteins separately. To ensure efficient cotransduction of cells with both AAVrh8 vectors, we selected bilateral thalamic injection as our delivery route, currently in clinical trials (ClinicalTrials.gov NCT04669535) (16, 17). Because AAV9-Bic_HexA/HexB encodes both *HEXA* and *HEXB*, here we investigated efficacy after CSF delivery, omitting the invasive thalamic injection required with use of the dual monocistronic vectors approach. AAV delivered to the CSF through either the lateral ventricle or the CM has been reported to achieve widespread CNS gene transfer in mice, cats, dogs, and nonhuman primates (26–32). This delivery approach has yielded impressive results for neuronopathic lysosomal storage diseases in which the AAV-encoded transgene is a secreted soluble enzyme that can be subsequently taken up by nontransduced cells via the mannose-6-phosphate receptor on the cell surface (29, 33–39). We are the first to combine all CSF delivery routes, to our knowledge, and we identified clear superiority of bilateral ICV injection combined with CM and LIT delivery using an intrathecal catheterization technique (40) when compared with intrathecal catheter delivery alone at the same dose. We previously showed that ICV injection combined with thalamic injection in sheep was insufficient to distribute to the spinal cord (19). We also evaluated CM injection alone using a blue dye or AAV9_GFP and found reduced staining in the lumbar region as compared with the cervical region, which is further indicative of reduced distribution in the spinal cord of the sheep (40). Therefore, combined LIT and CM injection was incorporated to enhance spinal cord transduction. This same route of injection was used in a phase I/II studies using monocistronic vectors for TSD and Sandhoff disease (17, 40).

We hypothesize that the added benefit of ICV injection is due to AAV9-Bic_HexA/HexB leakage through the ependymal cell layer and along the ICV needle tracks into the overlying parietal cortex, followed by retro- or anterograde AAV transport into the deep brain structures after CSF_ICV_CM_LIT injection (41, 42). Based on HexA enzymatic assays and vector genome biodistribution, we are unable to determine how thoroughly the AAV is distributed to the thalamus and striatum, because assays from homogenates can only inform about the whole structure. However, GM2 storage data and immunofluorescence in thalamus and striatum indicate dramatic improvement but some degree of incomplete correction. Future studies may suggest thalamic injections may be required to achieve complete disease correction. The difference between the AAV_CSF_CM-LIT cohort and the AAV_CSF_ICV-CM-LIT cohort, as measured by vector genomes, appears less pronounced than as measured by HexA activity. One could speculate that the slightly higher vector genome levels in the AAV_CSF_ICV-CM-LIT cohort are sufficient to drive enzyme expression higher in forebrain areas where projections to other areas are critical for therapeutic effect. One could consider increasing the dose in the AAV_CSF_CM-LIT cohort to transduce more cells in these regions; however, when scaled up for the human brain, doses would exceed that of 1 × 10^15^ vg in patients, and higher doses have been associated with toxicities and even death of patients (7, 43–48).

As we have described, the AAV_CSF_ CM_LIT injection was selected to treat the hindbrain and spinal cord. In the TSD+AAV_CSF-CM-LIT cohort, 1 sheep did better than expected (39 months old), suggesting individual variability between animals. The primary disease sign observed in these sheep was a proprioceptive ataxia that localizes on neurological exam to any upper motor neuron in the CNS; therefore, reduced distribution to the forebrain could account for the worse performance in gait and ambulation. An inability to walk triggers humane euthanasia; therefore, the TSD+AAV_CSF-CM-LIT cohort had a shorter survival. Furthermore, the maze test also relies on a functional forebrain for navigation; therefore, we hypothesize that reduced distribution to the cerebral cortex and deep brain structures would have contributed to poor performance in the maze.

Long-term biomarker data derived from CSF (i.e., HexA activity and GM2 ganglioside levels) suggests waning of the AAV9-Bic_HexA/HexB effect several years after gene transfer, which is discordant with HexA activity and GM2 ganglioside levels analyzed from tissue. CSF HexA levels slightly trended downward and GM2 trended upward over time in individual animals, but no difference was noted in enzymatic activity or vector genomes in animals sacrificed at 5 months or at 4 years after gene transfer. Similar findings have been reported in CSF collected over 7 years in WT dogs after CSF AAV9 administration; loss of transgene expression in CSF was evident but inflammatory/immune reactions were ruled out as an etiology (49). Current analysis of CSF biomarkers is the only patient-compatible method to assess changes in the neurochemistry of the CNS, but these data are a cautionary tale about overinterpretation of that data in clinical trials. Undoubtedly, the increase in HexA enzyme activity and reduction in GM2 ganglioside levels linked to the impact on survival are a clear indication of the efficacy of the present intervention. CSF data are thought to be a summation of the global impact on the CNS physiology. However, the slow worsening of efficacy biomarkers from 4 months to 5 years with no changes in tissue-level outcomes (i.e., enzyme activity and AAV genome copies) suggests that changes in the physiology of cells in direct contact with CSF (namely, ependymal cells, choroid plexus epithelial cells, and leptomeningeal cells) may have an oversized effect on biomarkers in this biofluid. This may be explained by slow turnover of these cell populations and resulting loss of AAV vector genomes. The turnover rate of mouse ependymal cells is 18 months (50), while the rate of choroid plexus (CP) epithelial cell replacement is suggested to be low (51), but we were unable to find a study in which this has been rigorously evaluated. The observation that CP ependymal cells have neural stem cell properties, or that neural stem cells are interspersed with ependymal cells in CP, makes determination of turnover rate considerably more challenging (52). The turnover of these populations in higher species is largely unknown, and in this study, or any other to our knowledge, the presence of AAV vector genomes over time was not analyzed. Further studies will be necessary to address this possibility directly.

Turnover of glia could be an alternative explanation for the slow change in CSF biomarkers, given the turnover rate of this CNS population (53); however, we did not find this in the in situ hybridization studies. It is possible that small changes below our level of detection may be masked by experimental variability associated with animal experiments or related to the sampling of CNS tissues that, although exhaustive, is also a composite of large numbers of cells. Nonetheless, it is important to note that the AAV9-treated TSD sheep remained healthy at 5 years of age. This information should be considered in future clinical trials in which CSF biomarkers are important outcome measures, but the biomarkers must be contextualized among others such as MRI or MR spectroscopy and clinical assessment to develop an objective measure of impact in patients.

Low HexA expression in the peripheral organs in both the i.v.- and CSF-treated groups could be due to age of treatment or difference in tissue expression driven by the promoter in this bidirectional AAV. The TSD+AAV_IV cohort received gene therapy at 1–2 weeks of age, whereas the TSD+AAV_CSF_ICV-CM-LIT or TSD+AAV_CSF_ CM-LIT cohorts were treated at 3 weeks of age. It is well established that neonatal treatment of liver results in dilution of the AAV genomes due to rapid hepatocellular division during growth of the animal (54, 55). Lack of kidney expression is consistent with findings by other groups in which AAV gene therapy of kidney was reported to be inefficient (56). The qPCR analysis demonstrated that AAV vector genomes are present in kidney for both i.v.- and CSF-treated cohorts (Supplemental Figure 2C), and lack of HexA activity suggests the promoter is not functional in this tissue. In cardiac muscle, the TSD+AAV_IV cohort trended toward greater transduction (vector genome) compared with the CSF-treated cohort, but HexA expression in this tissue was slightly lower than expected based on vector genome biodistribution. This also may also be a feature of promoter efficiency in cardiac tissue. Spleen and lung had modest amounts of vector genomes present, again with no HexA expression or storage clearance (Figure 5, C and D). We also attribute the lack of expression to limited promoter activity in this tissue. Skeletal muscle is the exception to this finding; HexA expression was highest in skeletal muscle in the TSD+AAV_IV cohort, and there was a trend toward lower levels in the cohorts that received CSF delivery. Because skeletal muscle mass increases during postnatal development through a process of hypertrophy, vector genomes are not lost over time in this tissue (57). We have seen similar results in AAV-driven RNA expression in the muscle of a cow after i.v. gene therapy using a similar bidirectional AAV design (58). In general, GM2 storage in peripheral organs is approximately 100-fold less than in the CNS (Figure 5, B and D). Because peripheral organ disease is not the cause of death in TSD or Sandhoff disease, this might not have high clinical significance. However, peripheral organ disease may become a larger problem in patients with extension of their life after receiving CNS-directed AAV gene therapy.

Here we report the most efficacious treatment for TSD and Sandhoff disease in the sheep model to date. We achieved broad biodistribution and partial correction of deep brain structures without direct injection into the brain parenchyma, and we conclude that this was provided by the contribution of bilateral ICV injection. Planned clinical trials will be based on this dose, scaled up by brain weight, and the predicted human dose is approximately 1 × 10^15^ vg. Additionally, based on data generated here, we plan to treat patients by combined bilateral ICV, CM, and LIT delivery, because lateral ventricular access is routine in pediatric and adult patients and we have established safety using catheter-mediated CM and LIT delivery in ongoing clinical trials.

## Methods

### Sex as a biological variant

This study includes both male and female sheep to account for sex as a biological variable.

### Animals and sample collection

All animal procedures were conducted in accordance with the guidelines of University of Massachusetts Medical School IACUC. The Jacob sheep colony was donated from a farm in Texas. CSF and blood samples were collected from all sheep at the time of surgery, MRI, and the humane endpoint. Jacob sheep were euthanized using a 150 mg/kg sodium pentobarbital overdose. Brain, spinal cord, and peripheral tissues were collected for storage at either –80°C or in 10% neutral-buffered formalin. Sheep genotypes were confirmed by PCR amplification of genomic DNA (DNeasy Blood and Tissue Kit; QIAGEN) extracted from blood.

### AAV vectors

The bicistronic AAV9 vector carried an expression cassette comprising a central CMV enhancer flanked by 2 chicken β-actin promoters in opposite directions, with no introns. The sheep HexA subunit is flanked by an SV40 poly(A) signal, while the sheep HexB subunit is flanked by an RBG poly(A) signal. AAV vectors were produced by triple transfection, as previously described, and purified by iodixanol gradient ultracentrifugation (59). The formulation was then dialyzed in Dulbecco’s PBS without calcium and magnesium (Thermo Fisher Scientific). The vector consisted of greater than 90% full capsids. Vector titration was targeted to the SV40 polyA (forward primer, 5′-AGCAATAGCATCACAAATTTCACAA-3′; reverse primer, 5′-CCAGACATGATAAGATACATTGATGAGTT-3′; probe, 56-FAM/AGCATTTTTTTCACTGCATTCTAGTTGTGGTTTGTC/36-TAMSp).

### Treatment groups

This study includes both male and female sheep to account for sex as a biological variable. Untreated TSD (*n* = 15) and age-matched WT (*n* = 10) sheep and AAV-treated sheep (*n* = 17) were included in this study. Animals were sedated with 0.1 mg/kg midazolam, i.v. jugular catheters were placed, and vector was administered to TSD sheep (*n* = 5) by a bolus i.v. injection of AAV vectors to the jugular vein (5 × 10^13^ vg/kg) at 9–13 days of age (2–4 kg at the time of injection). Two cohorts of TSD sheep received CSF administration of AAV (1 × 10^14^ vg total dose) at 3 weeks of age. One cohort received 75% of the dose through bilateral ICV injection, 12.5% through the CM, and 12.5% through LIT injections (*n* = 9). Other cohort received 75% of the dose through CM and 25% through LIT injections (*n* = 3). The total volume varied between sheep and was between 8–20 mL. Examiners were not blinded to treatment group except for GM2 ganglioside analysis.

### CNS administration of AAV

Prior to brain surgeries, sheep were premedicated intramuscularly with buprenorphine (0.02 mg/kg), acepromazine (0.05 mg/kg), and glycopyrrolate (0.01 mg/kg). Sheep were anesthetized by i.v. midazolam (0.3 mg/kg) and ketamine (10 mg/kg), intubated, and anesthesia was maintained using isoflurane gas. Meloxicam (0.25 mg/kg) and buprenorphine extended release 0.27 mg/kg were administered subcutaneously for pain management.

#### AAV administration to CM and LIT.

Sheep were placed in left lateral recumbency with lower lumbar spine flexion, and injection to the LIT space was performed as described previously (40). The lumbar puncture was performed using a 17-G Tuohy spinal needle at the lumbosacral intervertebral space (L6/L7–S1) and 5–12 mL of CSF was collected. A 1.7-F outer diameter intravascular microcatheter with a 0.355 mm (0.014 inch) guidewire (SL-10, Stryker Neurovascular) was introduced into the subarachnoid space via the Tuohy needle. Under fluoroscopic guidance, the microcatheter was navigated into the CM or premedullary cistern with the “J”-shape wire tip advanced slightly distal to the catheter. Cone beam computed tomography (Allura Xper FD20 system) was used to confirm adequate catheter position relative to bony and neural structures. After confirming the catheter position, 1 mL of iodinated contrast (Omnipaque 240 mg/mL; GE Healthcare) was slowly injected to identify the distribution pattern of contrast material prior to vector injection. The Tuohy needle was removed over the wire and AAV was infused at rate of 1 mL/min at level of the CM. Prior to removal of catheter, AAV was infused at level of L4 at the rate of 1 mL/min.

#### AAV administration to lateral ventricles.

ICV injections were performed using a presurgical MRI and intraoperative neuronavigation. Briefly, to register the sheep head to the anatomic MRI, an array of fiducial markers (Rogue Research) was used as previously described (60). Six MRI-sensitive, adhesive-backed fiducials were adhered to the circular portion of each fiducial peg. The fiducial markers were visualized as disk-shaped hyperintense marks on the MRIs, which were visualized with the Brainsight neuronavigation software (Rogue Research). To implant the fiducial array, the skin of the forehead was aseptically prepared. A midline skin incision approximately 5 cm long was made over the frontal bone and a post was attached to the frontal bone using ceramic screws and bone cement (eSutures.com). The fiducial marker array was then attached to the post prior the MRI. Anatomic MRIs were acquired in a Phillips 3T scanner (Phillips Ingenia 3T; Philips Healthcare) using an anterior coil (Philips Healthcare). The imaging protocol included 3D T1-weighted magnetization–prepared-rapid gradient echo sequence (repetition time/echo time [TR/TE] 10/5 ms; flip angle = 8°; number of averages [NSA] = 8; matrix = 268 × 268; slice thickness = 0.75 mm; field of view [FOV] = 200 × 200 mm).

After imaging, the fiducial array was removed, and the post was left in place with skin closed above. Images were transferred to Brainsight neuronavigation software for target identification and trajectory planning. For ICV injection, sheep were positioned in sternal recumbency with the head secured to a stereotaxic frame. The skin of the forehead was prepared aseptically, sutures were removed, and the fiducial array was reattached to the post. The fiducial array was used to register the patient using the Brainsight reference coordinate system, which uses an optical sensor camera and subject tracker with infrared reflective spheres mounted to the stereotaxic frame. Injection trajectories were planned to avoid the lateral ventricles and major blood vessels. After calculation of the skull thickness, a 2–3 mm hole was hand-drilled in the skull as the entry points for targeting lateral ventricles. Backflow of CSF was used to confirm the location in the right lateral ventricle. The AAV injection to lateral ventricles was performed off needle at the rate of 200 μL/min. In the cohort of sheep that received ICV, CM, and LIT administration of AAV, injections to CM and LIT were performed first and then surgery continued for ICV injections. There was 1 hour window (approximately) between CM/LIT and ICV injections.

### MRI

Sheep were noninvasively monitored, using different modalities of MRI, for up to 3 years after AAV administration. Untreated TSD sheep were imaged at the humane endpoint.

#### T1- and T2-weighted MRIs.

The imaging protocol for T1-weighted anatomic MRIs was described in *AAV administration to lateral ventricles*. The imaging protocol for 2D T2-weighted MRI included TR/TE 3,000/80 ms; flip angle = 90°; NSA = 8; matrix = 220 × 179; slice thickness = 1.75 mm; FOV = 120 × 120 mm).

#### MRS and analysis.

MR spectra were obtained using a single-voxel spectroscopy STEAM sequence in parietal cortex, thalamus, and cerebellum after shimming. Scan parameters included 12 × 12 × 15 mm^3^ voxel size, TR = 2,000 ms, TE = 9 ms, NSA = 192, and spectral bandwidth of 2,000 Hz. The LCModel basis set for Philips was used for analysis (https://www s-provencher.com/pages/lcmodel.shtml). Standard deviations higher than 30% were excluded. The following metabolites were examined: summed NAA plus NAAG (a neuronal integrity marker), Cr plus PCr (brain energy metabolism and potential gliosis marker), and GPC plus PCh (a marker of neuronal membrane turnover). Each metabolite concentration was normalized to the average of that metabolite concentration in the WT sheep cohort collected at the same time point and plotted.

#### DTI and analysis.

Diffusion images were acquired using a 2D echo-planar imaging diffusion sequence. DTI data were collected at a b-value of 1,000 s/mm^2^ with 48 diffusion directions. DTI was acquired using the sequence with a voxel size of 2 × 2 × 2 mm^3^, TR = 6,670 ms, and TE = 58 ms. The diffusion images were processed using the DSI Studio software package (June 22, 2016, version; http://dsi-studio.labsolver.org) using a region of interest–based (ROI-based) analysis method, as described previously (61). The quality of images was inspected before analysis and any image affected by motion or any other artifact was rejected. In DSI studio, diffusion MR images were converted into an SRC file. A brain mask was then conformed onto the source image and the mask was adjusted by alteration of a threshold to select the brain tissue precisely. Adjustment of the mask was completed on an individual basis and included removing external fragments as well as smoothing and dilating the mask as necessary. Upon finalizing the mask, the image was reconstructed into a FIB file using the DTI reconstruction method. ROIs in thalamus were defined. To draw the ROIs, voxels were selected manually to include the white matter within the target structure. A fiber-tracking algorithm was used within DSI Studio, and a seeding region was placed at whole brain in addition to the ROI. Within each region, a maximum of 10,000 tracts were analyzed with fiber length minimum/maximum of 30/300 mm, 60° angular threshold, and 1 mm step size. DTI scalars were calculated, including FA, apparent diffusion coefficient, axial diffusivity, and RD.

### Neurological examination

Standard veterinary neurological examinations including gait (cerebellar or proprioceptive ataxia), proprioception (abnormal placement of hooves), and strabismus (loss of eye coordination) assessments were performed on all cohorts. The neurological exams were scored using discrete numbers from 1 to 5, with increasing scores indicating worsening neurological signs. However, the clinical rating score was calculated by taking the average of all scores; therefore, clinical rating scores contain decimal numbers.

### Hexosaminidase activity

Hexosaminidase activity was measured in blood, CSF, and tissues, as described previously (18). Tissues were lysed in 0.1% Triton X-100 in 0.01 M phosphate citrate buffer pH 4.4 using a TissueLyser II (QIAGEN) with 5 mm stainless steel beads at 20 Hz for 30 seconds with 3 pulses. Lysates underwent 3 freeze-thaw cycles alternating between a dry-ice ethanol bath and 37°C water bath.

### MRS quantification of GM2 ganglioside

GM2 quantification was done using liquid chromatography–tandem mass spectroscopy as previously described (62, 63). GM2 was extracted in the presence of d3-GM2(18:0) as an internal standard. A quality control sample was prepared by pooling a portion of study samples and injected every 10 study samples to monitor instrument performance. Relative quantification data were reported as peak area ratios of the GM2 to their internal standards. Final concentrations are reported as nanograms per gram tissues or micrograms per milliliter CSF.

### Biodistribution analysis

Vector genome copy numbers from CNS and peripheral tissues were determined by qPCR after extraction of total DNA using a DNeasy blood and tissue kit (QIAGEN). Vector genome content in each tissue was determined using 100 ng of total DNA by a qPCR method using primers and probes for the SV40 poly(A) in the vector (forward primer, 5′-AGCAATAGCATCACAAATTTCACAA-3′; reverse primer, 5′-CCAGACATGATAAGATACATTGATGAGTT-3′; probe, 56-FAM/AGCATTTTTTTCACTGCATTCTAGTTGTGGTTTGTC/36-TAMSp). The lower limit of detection in the qPCR assay was 100 genome copies/100 ng DNA, and any sample below this limit was considered nondetectable.

In situ hybridization (RNAscope) and image acquisition OCT-embedded blocks were cut into 10-μm sections. Sample preparation for RNAscope staining was performed following the manufacturer’s guidelines. An RNAscope probe was designed (RNAscope Probe BiCb6-No-XOaBt-C1 to target the CMV region sequence in the AAV9 HexA/HexB bicistronic expression cassette. RNAscope Negative Control Probe – DapB (catalog 310043) was used as a negative control. In situ hybridization to detect probe signal was performed using the RNAscope Multiplex Fluorescent Reagent Kit, version 2 (catalog 323100) and TSA Vivid Fluorophore 520 (PN 323271), following the manufacturer’s instructions. Imaging was performed using a Leica Microscope (Leica DMi8 Thunder imager) for ×20 and ×40 magnifications. For the base scope assay, the BaseScope Probe - BA-SV40polyA-2zz-st1-C1 (catalog 1556641-C1), which is designed to target the SV40 poly(A) region in the AAV9 HexA/HexB bicistronic expression cassette, and BaseScope Negative Control Probe - DapB-3ZZ (catalog 701011) were used as negative controls. Both were purchased from ACDbio. The in situ hybridization was performed using BaseScope Reagent Kit v2 RED (ACDbio, 323900), following the manufacturer’s instructions. Staining was visualized under bright-field microscope, and imaging was acquired with a Leica DMi8 Thunder imager microscope (Leica Microsystems) at ×20 and ×40 magnifications. Images was analyzed with LAS X software.

### Immunostaining and image acquisition

Paraffin-embedded blocks were cut into 5-μm sections for immunostaining. Standard H&E staining was performed on deparaffinized and rehydrated slides for histopathologic analyses. Bright-field images were captured using a Leica DMi8 inverted microscope (Leica Microsystems). Primary antibodies used for immunofluorescence included MAP2 (CH22103, Neuromics), recombinant anti-Olig2 antibody (ab109186, Abcam), MBP (NB600-717, Novus Biologicals), calcium-binding adapter molecule 1 (CP290, Biocare), and glial fibrillary acidic protein (556329, BD Pharmingen). Secondary antibodies (all from Thermo Fisher Scientific) included donkey anti–rabbit IgG, Alexa Fluor Plus 647 (catalog A32795), donkey anti–mouse IgG Alexa Fluor Plus 555 (catalog A32773), donkey anti–rat IgG Alexa Flour Plus 555 (catalog A48270), and donkey anti–chicken IgG Alexa Fluor 555 (catalog A78949). Fluorescence images were captured using a Leica DM5500 B upright microscope (Leica Microsystems).

### NAb assay

NAb titers in blood and CSF were measured as described previously (64). Samples were heat inactivated at 56°C for 35 minutes. Recombinant AAV.CMV.LacZ (*n* = 109 genome copies/well) was diluted in serum-free DMEM and incubated with 2-fold serial dilutions (initial dilution, 1:20) of heat-inactivated serum samples on DMEM for 1 hour at 37°C. Subsequently, the serum-vector mixture was added to 96-well plates seeded with 1 × 10^5^ Huh7 cells/well that had been infected 2 hours earlier with WT HAdV5 (*n* = 50 viral particles/cell). After 1 hour, each well was supplemented with an equal volume of 20% FBS/DMEM and incubated for 18–22 hours at 37°C and 5% CO_2_. Then, cells were washed twice in PBS and lysed, and the lysate was developed with the mammalian-galactosidase assay kit for bioluminescence, in accordance with the manufacturer’s protocol (Applied Biosystems), and measured in a microplate luminometer (Clarity; BioTek). The NAb titer was reported as the highest serum dilution that inhibited AAV.CMV.LacZ transduction (β-galactosidase expression) by more than 50%, compared with the mouse serum control (Sigma-Aldrich, S3509).

### Statistics

Prism 8.0 (GraphPad Software) was used for statistical analysis. Significant difference was defined as *P* less than 0.05. Clinical rating scores for neurological deficits and neurological scores were analyzed by Mann-Whitney tests, and effect was determined by a Hodges-Lehmann test. To perform statistical analysis on HexA, GM2, qPCR, DTI scalars and MRS, Gaussian distribution of each group of data was examined using the Shapiro-Wilk test. For non-Gaussian distribution, a nonparametric Kruskal-Wallis test followed by Dunn’s test was performed. For Gaussian distribution, Brown-Forsythe and Welch’s ANOVA tests, followed by Dunnett’s T3 test, were performed. If cohorts showed Gaussian distribution with equal standard deviation, 1-way ANOVA was performed for statistical comparison, followed by the recommended multiple comparison using GraphPad Prism software. To perform correlation analysis of DTI with GM2 concentrations in CSF, normal distribution of data was investigated using the Shapiro-Wilk test. Correlation analysis for data with normal distribution was performed using Pearson’s test. For non-normal distribution, Spearman’s correlation analysis was used. A 2-tailed *P* value determined the significance of correlation.

### Study approval

All animal procedures were conducted in accordance with the guidelines of University of Massachusetts Medical School IACUC.

### Data and materials availability

The data supporting the findings of this study are available within the article, the Supporting Data Values file, or from the corresponding author upon reasonable request.

## Author contributions

HGE designed the studies and oversaw the experiments and downstream analysis. TT performed AAV plasmid production and purification for CSF studies, MRI and analysis, and performed immunohistochemistry. DF and JG performed sheep genotyping. KLM and HGL performed AAV plasmid production and purification for IV studies. DRM and AG performed IV sheep administration and assisted in data interpretation. HGE, VA, and AP performed sheep CNS surgeries. RK and MJG provided help with running fluoroscopy during sheep surgeries. TT and SE performed the enzyme assays. ARB performed titration of AAV vectors for CSF studies. HGE, SB, SJ, AT, WCB, JG, HRB, AB, ED, LB, ST, RG, and RP provided veterinary care for sheep and performed behavioral studies. JG analyzed the behavioral data. TT, DF, HGE, JG, WCB, HRB, EH, YL, AM, and KLM performed sheep necropsies. TT, JTM, and SP performed tissue preparation and microscopy. XJ performed GM2 quantifications. TT, YL, EH, SM, and SE performed biodistribution studies. YL perfumed RNAScope and Base scope and imaging. MSE designed the bicistronic vector. GG, JX, and QS performed cell transfection and packaging. JK is a board-certified veterinary pathologist who evaluated some sections. TT performed analysis of biochemical and MRI data. HGE and TT wrote the manuscript.

## Funding support

This work is the result of NIH funding, in whole or in part, and is subject to the NIH Public Access Policy. Through acceptance of this federal funding, the NIH has been given a right to make the work publicly available in PubMed Central.

- NIH grant K08NS096219.
- National Tay-Sachs and Allied Diseases Association.
- UMass Chan Medical School.
- Scott-Ritchey Research Center.

## Supplementary Material

Supplemental data

Supplemental video 1

Supplemental video 2

Supplemental video 3

Supplemental video 4

Supplemental video 5

Supplemental video 6

Supplemental video 7

Supplemental video 8

Supporting data values

## Figures and Tables

**Table 1:**
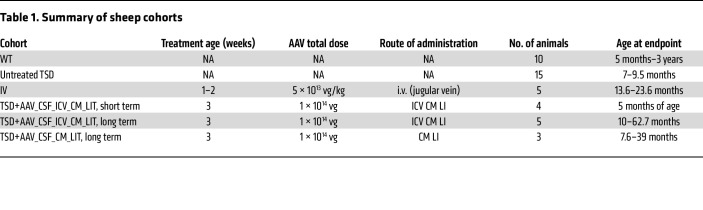
Summary of sheep cohorts

## Version 1 09/30/2025

In-Press Preview

## Version 2 12/01/2025

Electronic publication
