# Supplementary material for: Five-year analysis of efficacy and safety of a bidirectional AAV gene therapy in Tay-Sachs sheep
Source: J Clin Invest. 2025 Sep 30;135(23):e182942. doi: 10.1172/JCI182942 (PMC12646665; doi:10.1172/JCI182942)
Supplement: Supplemental data [file jci-135-182942-s166.pdf]

Supplementary Materials:

Supplemental Figure 1 to 10.

Supplemental Captions for Videos 1 and 2.

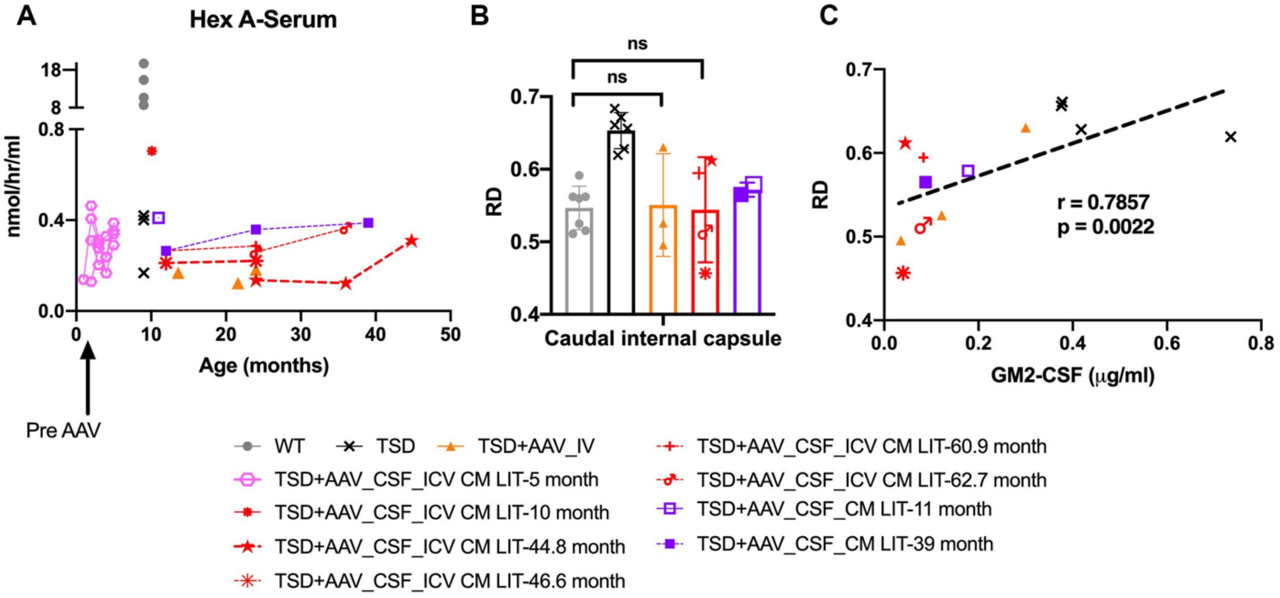

Supplemental Figure 1: Serum HexA and Radial Diffusivity as biomarkers of efficacy. A. HexA in serum over the lifespan of AAV treated sheep compared to normal and TSD control sheep. **B.** Radial Diffusivity (RD) in the caudal internal capsule. Kruskal-Wallis test followed by Dunn's multiple comparisons was performed for statistical analysis. For DTI analysis, each sheep imaged one time at the endpoint and DTI analysis was performed to determine RD. **C.** Correlation of RD with GM2 measured in CSF (p=0.0022). Correlation analysis was performed by Spearman test.

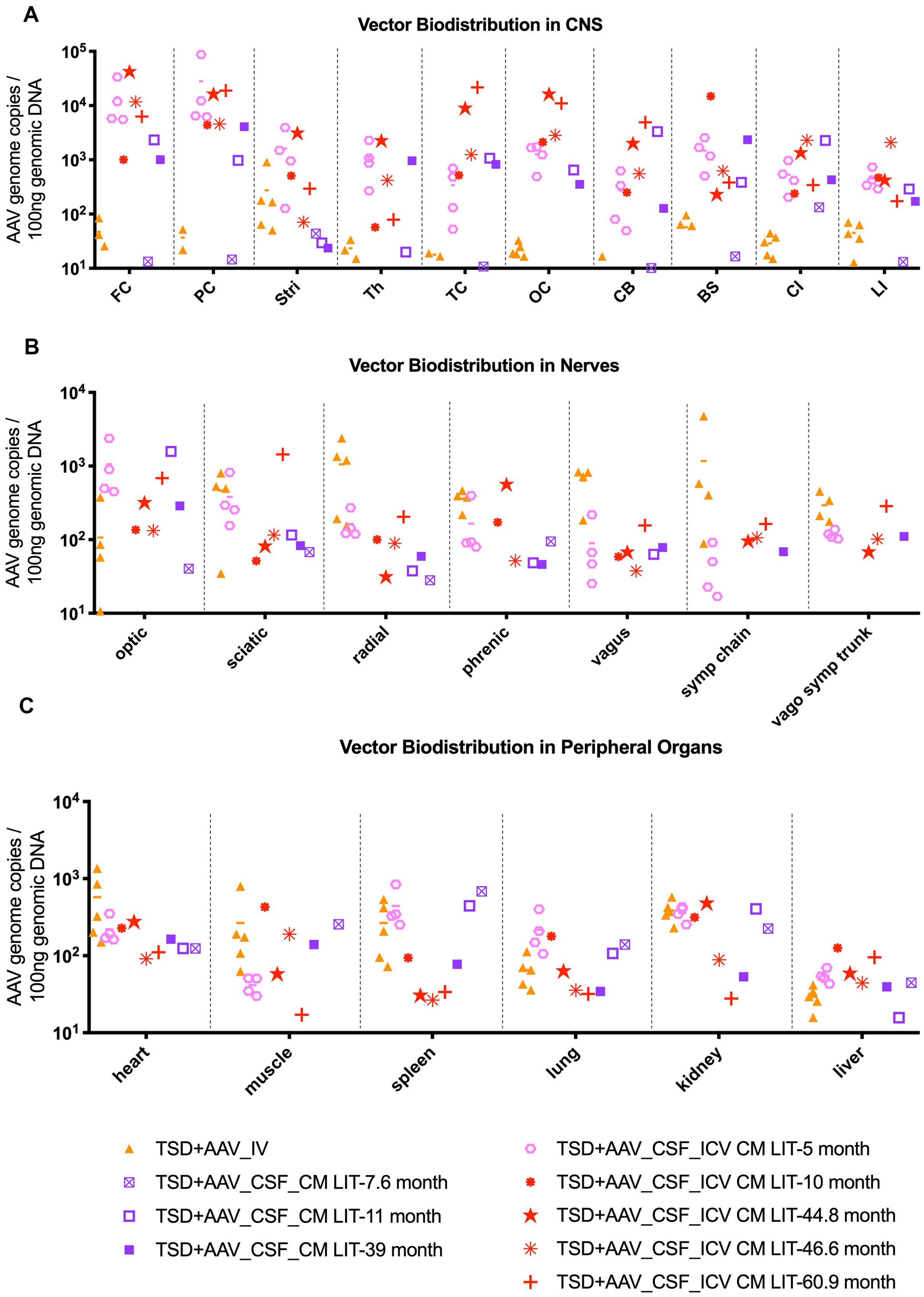

A
Vector Biodistribution in CNS
AAV genome copies / 100ng genomic DNA
10^5
10^4
10^3
10^2
10^1
FC
PC
Stri
Th
TC
OC
CB
BS
CI
LI
B
Vector Biodistribution in Nerves
AAV genome copies / 100ng genomic DNA
10^4
10^3
10^2
10^1
optic
sciatic
radial
phrenic
vagus
symp chain
vago symp trunk
C
Vector Biodistribution in Peripheral Organs
AAV genome copies / 100ng genomic DNA
10^4
10^3
10^2
10^1
heart
muscle
spleen
lung
kidney
liver
TSD+AAV_IV
TSD+AAV_CSF_CM LIT-7.6 month
TSD+AAV_CSF_CM LIT-11 month
TSD+AAV_CSF_CM LIT-39 month
TSD+AAV_CSF_ICV CM LIT-5 month
TSD+AAV_CSF_ICV CM LIT-10 month
TSD+AAV_CSF_ICV CM LIT-44.8 month
TSD+AAV_CSF_ICV CM LIT-46.6 month
TSD+AAV_CSF_ICV CM LIT-60.9 month

16

Supplemental Figure 2: Biodistribution of AAV in sheep. A. CNS biodistribution of AAV9 in the frontal cortex (FC), parietal cortex (PC), thalamus (Th), temporal cortex (TC), occipital cortex (OC), cerebellum (CB), brainstem (BS), cervical intumescence (CI) and lumbar intumescence (LI). Short term TSD+AAV_CSF_ICV-CM-LIT cohort (pink hexagon) had significantly higher vg in FC, PC and OC as compared to TSD+AAV_IV cohort. **B.** Optic nerve in short term TSD+AAV_CSF_ICV-CM-LIT cohort (pink hexagon) had significantly higher vg as compared to TSD+AAV_IV cohort **C.** No significant difference between vg of short term TSD+AAV_CSF_ICV-CM-LIT cohort (pink hexagon) and TSD+AAV_IV cohort in analyzed peripheral organs. Kruskal-Wallis test followed by Dunn’s multiple comparisons was performed for statistical analysis in all panels. Assays were repeated at least three times. Abbreviations: Symp. Chain (sympathetic chain). Vago symp trunk (vagosympathetic trunk).

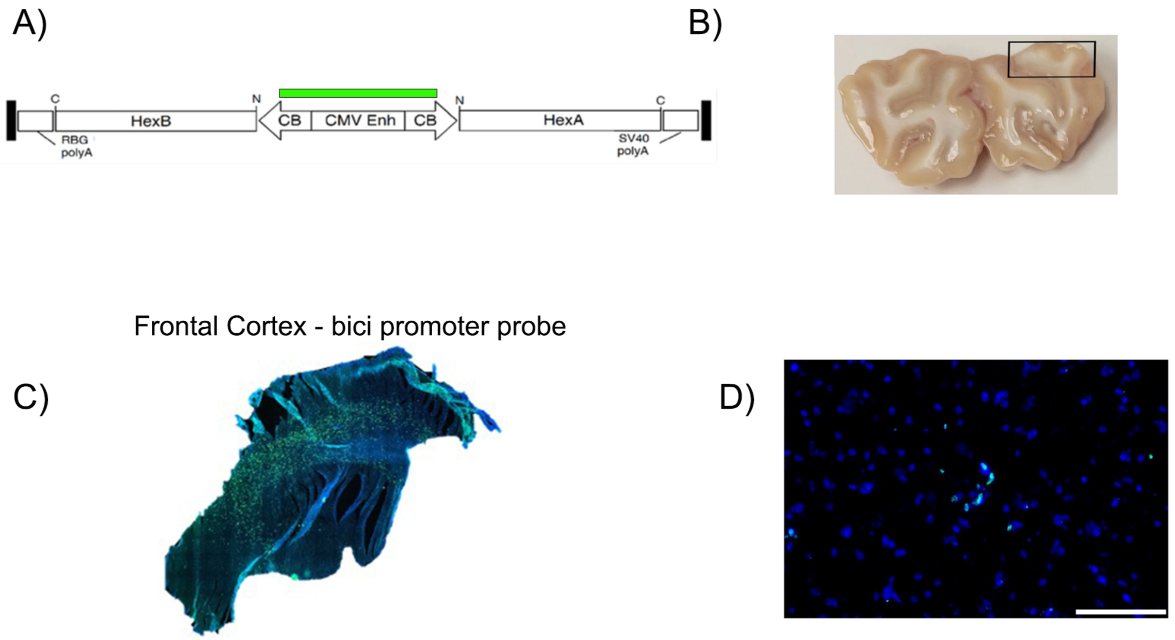

Supplemental Figure 3. Assessment of vector biodistribution in frontal cortex of 60.9-month-old sheep. A. RNAscope to the bici promoter (green)**. B.** Representative image of sheep frontal cortex. **C-D.** Vector biodistribution in long term treated sheep TSD+AAV_CSF_ICV-CM-LIT. Scale bar, 100 μm. Assays were repeated at least three times.

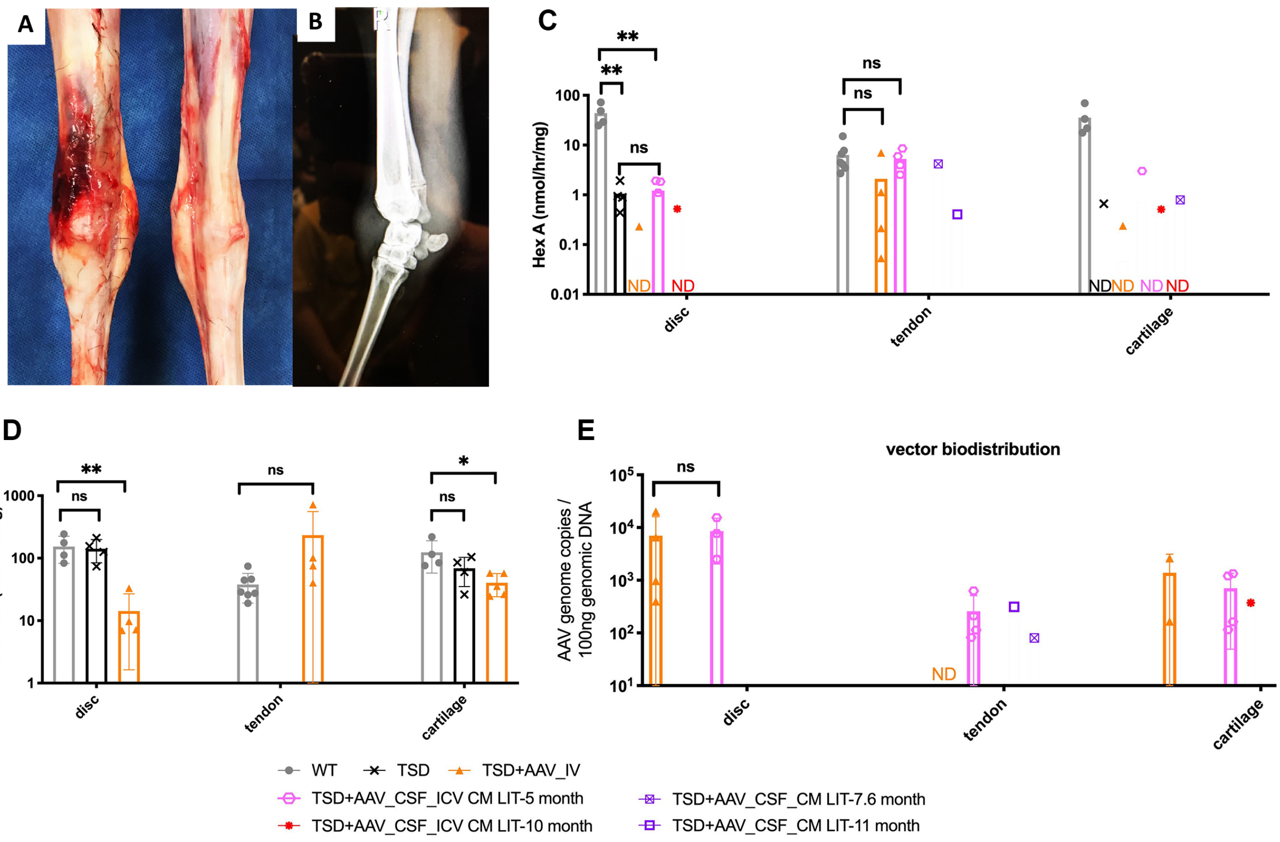

Supplemental Figure 4. Off target assessment in tendons and ligaments: A-B. Tendon injuries in TSD+AAV_IV treated cohort. **C.** Hex A activity in intervertebral disc, tendon, and cartilage (**P<0.009). **D.** total Hex activity in intervertebral disc, tendon, and cartilage (*P<0.028; **P<0.008). **E.** vector biodistribution in disc, tendon, and cartilage. Kruskal-Wallis test followed by Dunn's multiple comparisons was performed for statistical analysis in all panels. Assays were repeated at least three times.

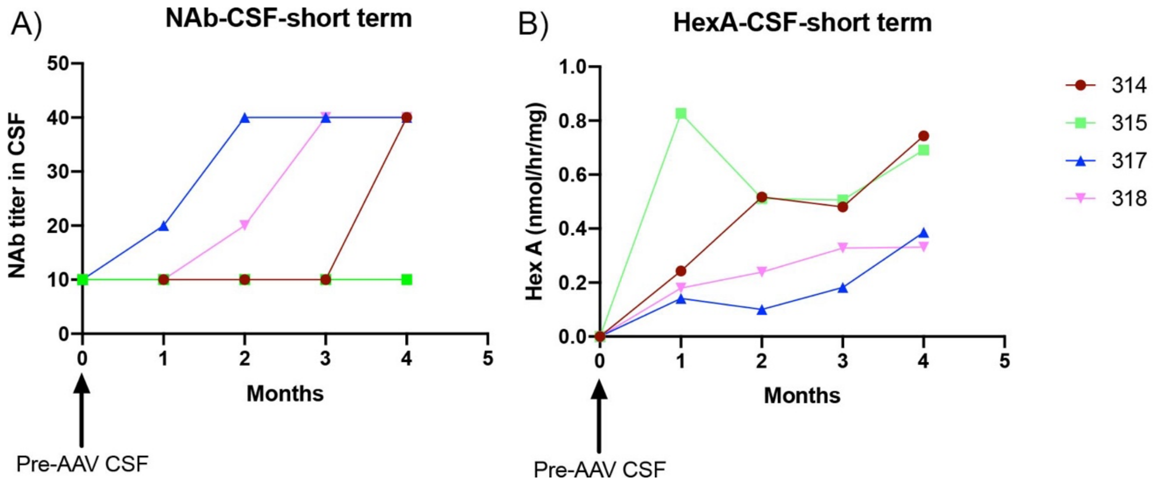

Supplemental Figure 5: Neutralizing antibody titer and HexA levels in CSF of individual sheep in the short-term treated TSD+AAV_CSF_ICV-CM-LIT cohort. A. Nab titers against AAV9 before and up to 4 months after CSF administration of AAV in CSF. **B.** HexA levels before and up to 4 months after CSF administration of AAV in CSF. The 4 sheep in the short term cohort are represented by pink hexagon in the HexA, GM2, qPCR plots.

71

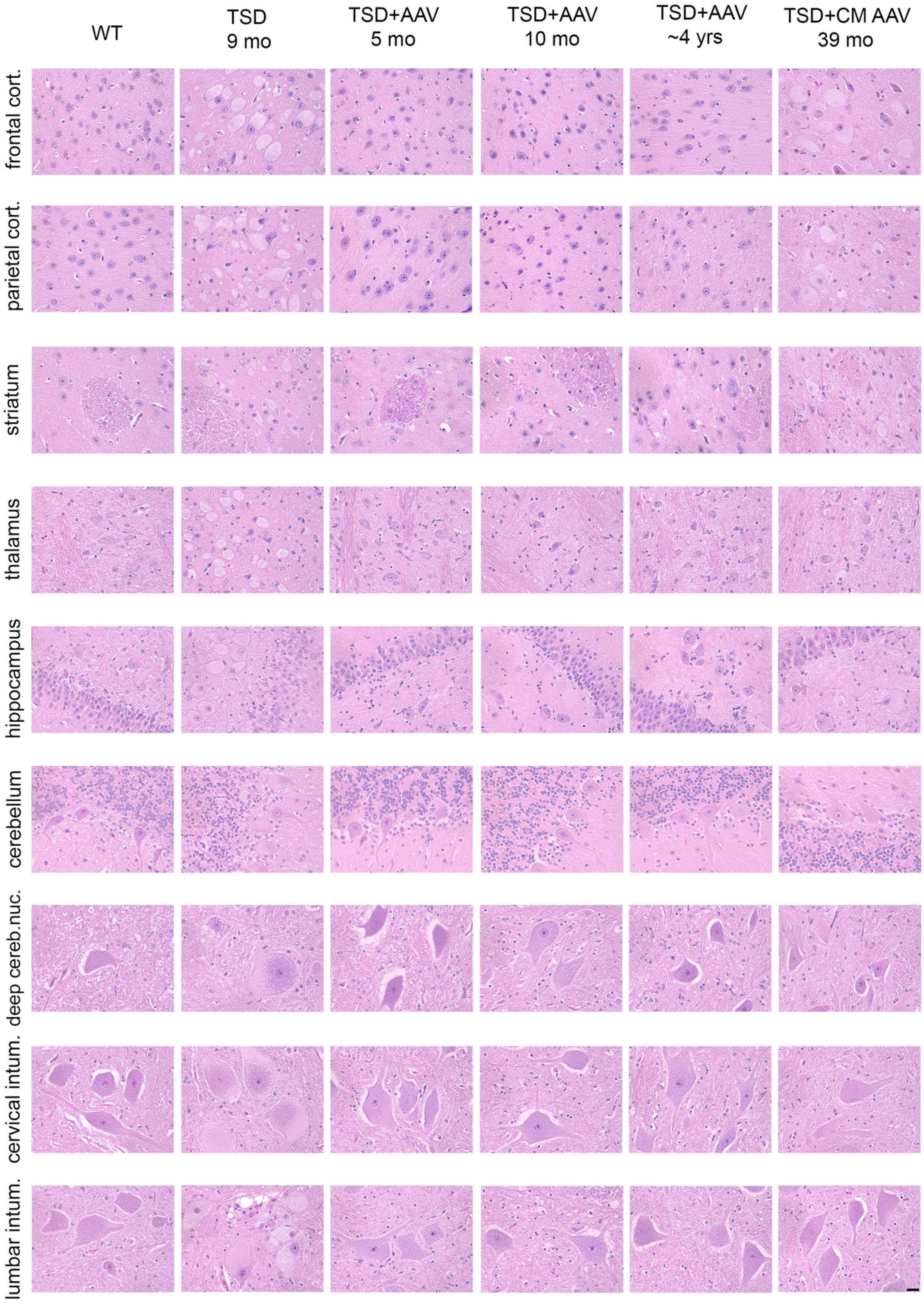
WT
TSD 9 mo
TSD+AAV 5 mo
TSD+AAV 10 mo
TSD+AAV ~4 yrs
TSD+CM AAV 39 mo
frontal cort.
parietal cort.
striatum
thalamus
hippocampus
cerebellum
deep cereb.nuc.
cervical intum.
lumbar intum.

Supplemental Figure 6: Hematoxylin and eosin–stained (H&E) sections of brain for all cohorts of sheep in the study. Morphological improvement noted in all treated groups throughout the CNS despite animals reaching humane endpoint. Scale bar, 25 μm.

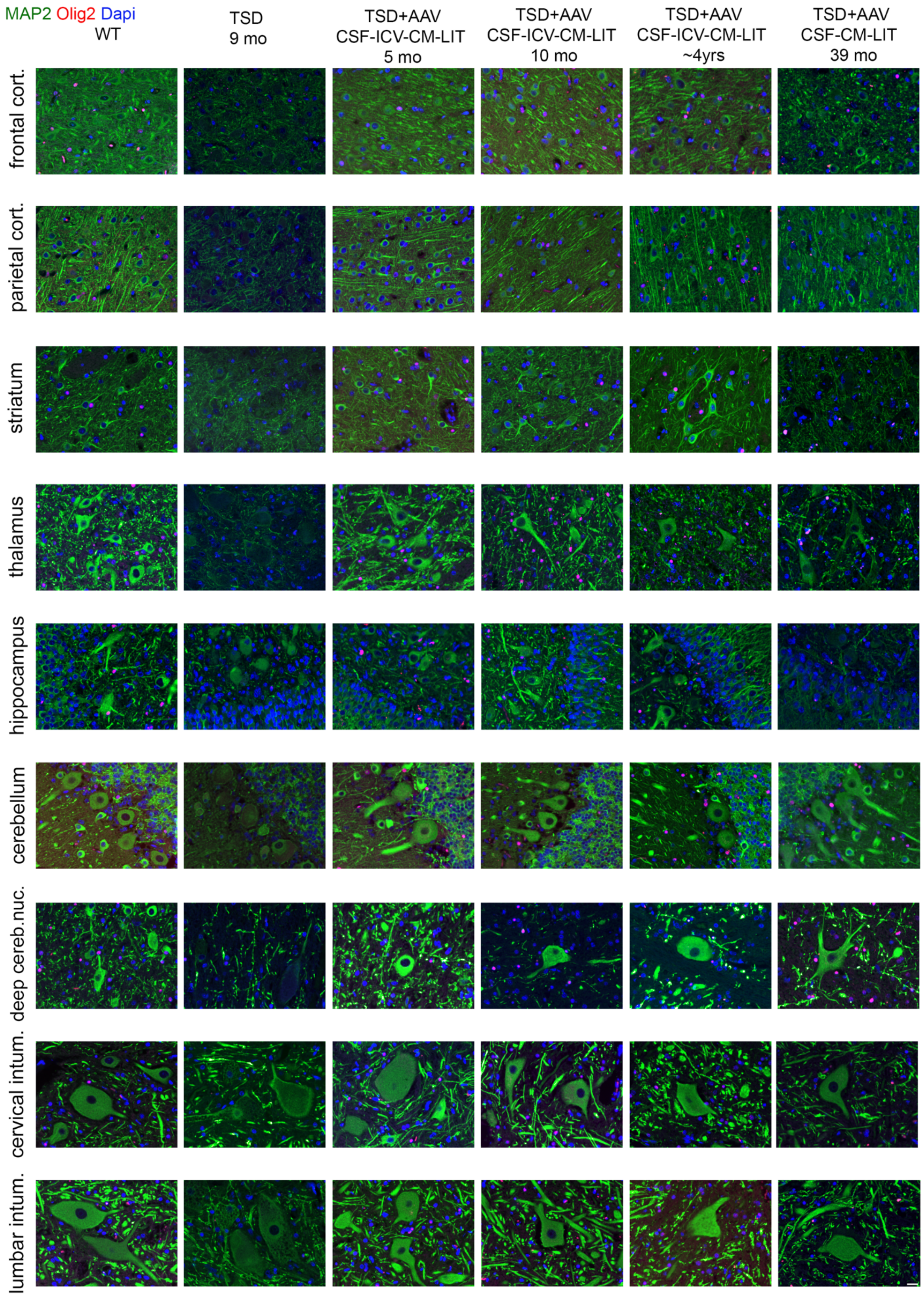
MAP2 Olig2 Dapi
WT
TSD
9 mo
TSD+AAV
CSF-ICV-CM-LIT
5 mo
TSD+AAV
CSF-ICV-CM-LIT
10 mo
TSD+AAV
CSF-ICV-CM-LIT
~4yrs
TSD+AAV
CSF-CM-LIT
39 mo
frontal cort.
parietal cort.
striatum
thalamus
hippocampus
cerebellum
deep cereb.nuc.
cervical intum.
lumbar intum.

Supplemental Figure 7: Neuronal and Oligodendrocyte immunofluorescence of all analyzed CNS sections. In addition to brain regions shown in Figure 6, this figure illustrates images of additional evaluated brain regions. MAP2, Olig2 and Dapi staining in various regions of brain and spinal cord. Scale bar, 20 µm.

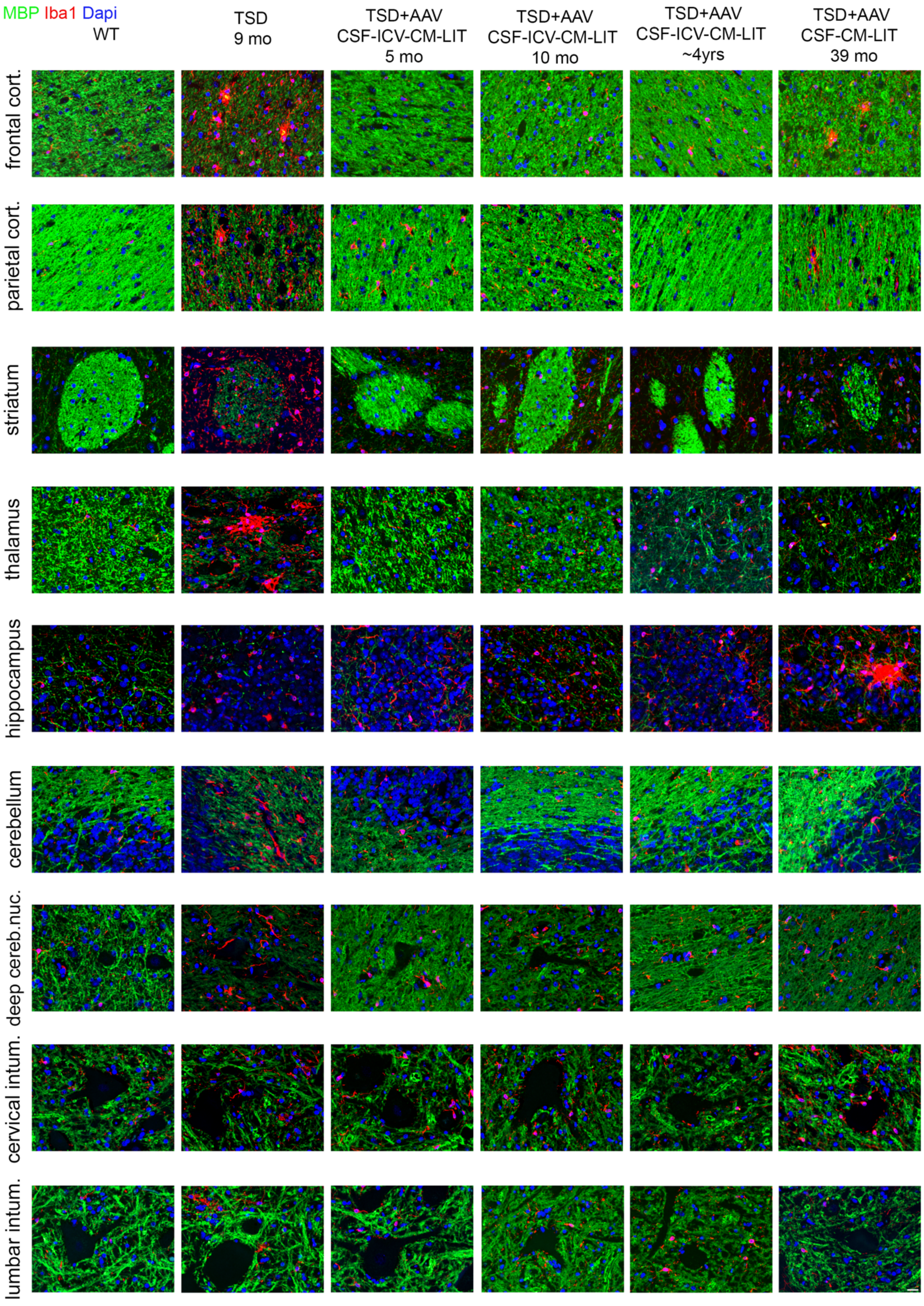

MBP Iba1 Dapi
WT
TSD
9 mo
TSD+AAV
CSF-ICV-CM-LIT
5 mo
TSD+AAV
CSF-ICV-CM-LIT
10 mo
TSD+AAV
CSF-ICV-CM-LIT
~4yrs
TSD+AAV
CSF-CM-LIT
39 mo
frontal cort.
parietal cort.
striatum
thalamus
hippocampus
cerebellum
deep cereb.nuc.
cervical intum.
lumbar intum.

82

Supplemental Figure 8: Myelin and microglial immunofluorescence of all CNS sections. In addition to brain regions shown in Figure 7, this figure illustrates images of additional evaluated brain regions. MBP, Iba1 and Dapi staining in various regions of brain and spinal cord. Scale bar, 20 µm.

87

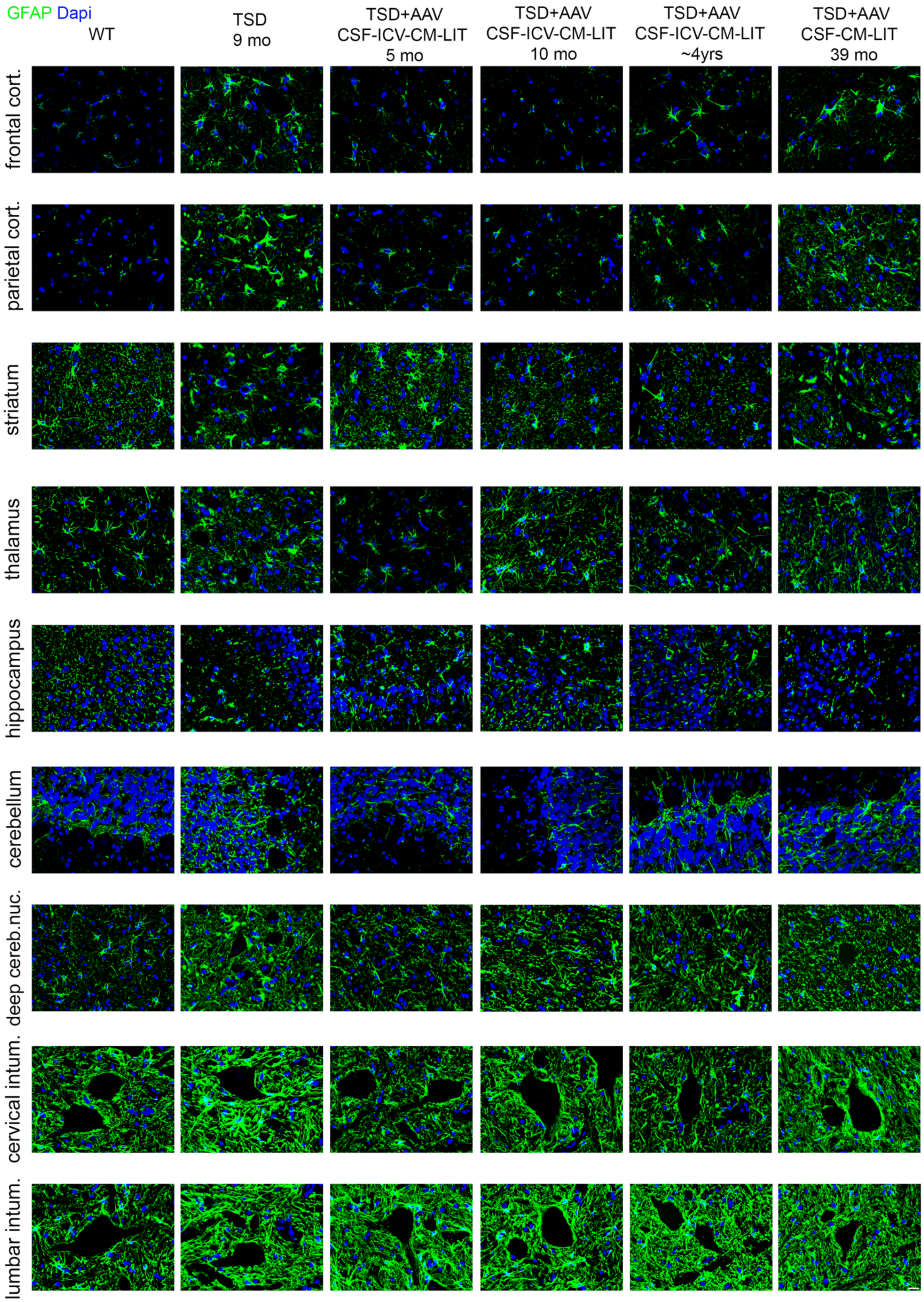

GFAP Dapi
WT
TSD
9 mo
TSD+AAV
CSF-ICV-CM-LIT
5 mo
TSD+AAV
CSF-ICV-CM-LIT
10 mo
TSD+AAV
CSF-ICV-CM-LIT
~4yrs
TSD+AAV
CSF-CM-LIT
39 mo
frontal cort.
parietal cort.
striatum
thalamus
hippocampus
cerebellum
deep cereb.nuc.
cervical intum.
lumbar intum.

Supplemental Figure 9: Astrocyte immunofluorescence of all CNS sections. GFAP and Dapi staining in various regions of brain and spinal cord. Scale bar, 20 μm.

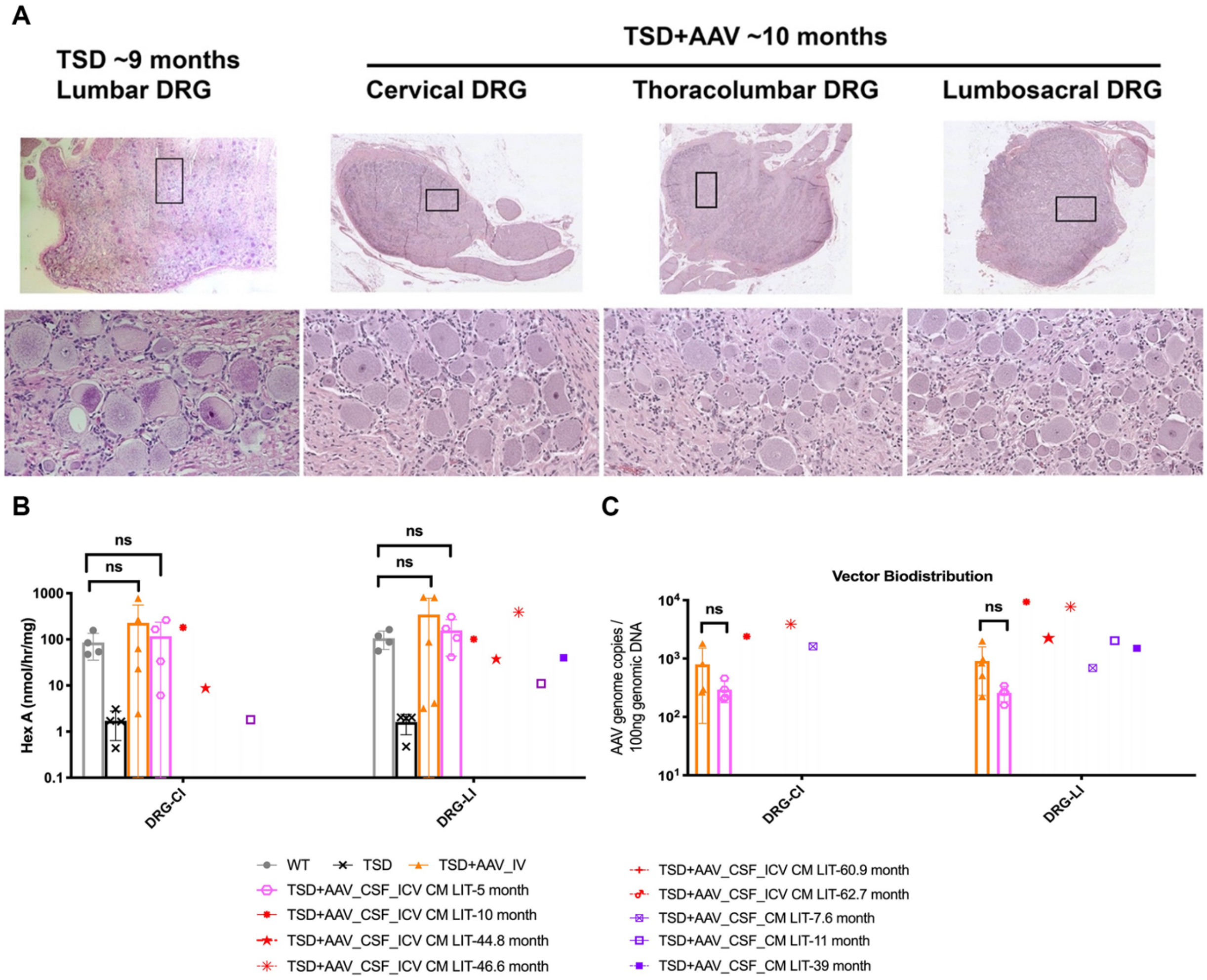

Supplemental Figure 10: DRG morphology, HexA activity and biodistribution A. H&E of DRG in 9-month-old TSD sheep as compared to 10-month-old TSD+AAV_CSF-ICV-CM-LIT sheep. **B.** HexA expression. **C.** vector biodistribution in DRGs at level of CI and LI of spinal cord. Ordinary one-way ANOVA with Sidak's multiple comparison were used for statistical analysis in B and C. Assays were repeated at least three times.

Supplemental Videos 1: Sheep gait assessment. A. WT sheep **B.** Untreated TSD sheep near endpoint **C.** TSD+AAV_CSF_ICV-CM-LIT 5 years after gene therapy #212. **D.** TSD+AAV_CSF_ICV-CM-LIT 4 years after gene therapy #271.

Supplemental Videos 2: Sheep in maze A. WT **B.** TSD **C.** TSD+AAV_IV **D.** TSD+AAV_CSF-ICV-CM-LIT
